# Altered brain network dynamics in motor functional neurological disorders: the role of the right temporo-parietal junction

**DOI:** 10.1038/s41398-025-03385-5

**Published:** 2025-05-15

**Authors:** Samantha Weber, Janine Bühler, Thomas A. W. Bolton, Selma Aybek

**Affiliations:** 1 Department of Neurology, Inselspital, Bern University Hospital, University of Bern, Bern, Switzerland; 2 University Hospital of Psychiatry Zurich, Department of Adult Psychiatry and Psychotherapy, 8032 Zurich, Switzerland; 3 Faculty of Medicine, University of Zurich (UZH), 8006 Zürich, Switzerland; 4 Translational Imaging Center (TIC), Swiss Institute for Translational and Entrepreneurial Medicine, 3010 Bern, Switzerland; 5 Danish Research Centre for Magnetic Resonance, Department of Radiology and Nuclear Medicine, Copenhagen University Hospital - Amager and Hvidovre, Copenhagen, Denmark; 6 Faculty of Science and Medicine, University of Fribourg, 1700 Fribourg, Switzerland; 7 Connectomics Laboratory, Department of Radiology, Centre Hospitalier Universitaire Vaudois (CHUV), 1011 Lausanne, Switzerland

**Keywords:** Diagnostic markers, Psychiatric disorders

## Abstract

Functional neurological disorders’ (FND) neuropathophysiology has been described as multi-network disturbances including aberrancies in the agency network highlighting the role of the right temporo-parietal junction (rTPJ). Refining the relevance of the rTPJ, we applied a co-activation pattern (CAP) based approach using the rTPJ as a seed in 58 patients with motor FND compared to 58 age- and sex-matched healthy controls (HC). Firstly, CAPs were derived from HC to identify functional alterations in the rTPJ network in FND patients. Secondly, motor subgroup characteristics in patients were examined using CAPs derived from the patient group. Compared to HC, patients were found to enter less frequently a state characterized by salience network and default mode network (DMN) co-activation along with executive control and somatomotor networks co-deactivation. Additionally, patients entered more often a state depicted by somatomotor-salience co-activation and DMN co-deactivation. Comparing motor subgroups, patients with functional weakness (FW) remained longer in a state characterised by salience and dorsal/ventral attention network co-activation and DMN co-deactivation compared to patients with no functional weakness (no-FW). FND patients overall exhibited a reduced coupling of the DMN and an increased coupling of the somatomotor network with the rTPJ compared to controls. Patient subgroups differed regarding coupling between the rTPJ and the attention network and DMN. rTPJ dynamic network alterations might reflect hampered flexibility in brain state switching and altered self-referential processes linked to impaired motor planning and execution, which seem to also differ between symptom types, indicating a potential phenotypic biomarker.

## Introduction

Patients with a functional neurological disorder (FND) present with heterogeneous symptoms including weakness, abnormal movements, dystonia, myoclonus or tremor which are not attributable to structural lesions of the nervous system [1]. Within the repeatedly sustained framework of a functional multi-network neuropathophysiology in FND [2], network alterations linked to an impaired sense of agency (SoA) have been highlighted [3]. The SoA can be described as the experience of being the active executor of an action [4], whereas the right temporoparietal junction (rTPJ) has been assumed to act as a key node in the comparator model, in which intention-based motor plans deriving from the supplementary motor area (SMA) and dorsolateral prefrontal cortex (DLPFC) are compared to actual motor outcome forwarded from the sensory system [5]. The SoA has been postulated to be disrupted in FND patients as they experience their symptoms being produced involuntarily, even though the involvement of voluntary corticospinal pathways is existing, observable in clinical examinations disclosing entrainment or distractibility [6]. Evidence from behavioral experiments sustains the hypothesis of distorted SoA [7–13]. Using functional magnetic resonance imaging (fMRI), the rTPJ was found to be hypoactive while patients experienced functional symptoms [14]. Further studies revealed hypoactivity [7, 13, 14] as well as hyperactivity [15, 16] in task-based fMRI targeting the SoA. Resting-state fMRI revealed increased functional connectivity (FC) from the rTPJ to sensorimotor regions [17], contrasted by findings of reduced FC from the rTPJ to limbic and sensorimotor feedback regions [14], to frontal regions [7, 18], SMA and insula [19], as well as the medial temporal lobe network [20]. In light of the inconsistency of available reports, the influence and relevance of the rTPJ network in FND patients, i.e., the FC from the rTPJ to other brain regions requires more attention and detailed investigation.

Two factors might account for the lack of homogenous findings in previous literature: firstly, the predominant use of *static* brain FC measures that are unable to capture underlying network fluctuations across the time-course of the fMRI measurement, and secondly, the diverse clinical characteristics of FND patients investigated.

On the one hand, while contemporary *static* FC approaches generally summarize the temporal correlations between a specified seed and other brain regions or networks [21] and consequently are insensitive to transient information, *dynamic* states of temporal characteristics in FC could also be expressed in co-(de)activation patterns between a specified seed region and other brain regions, which can account for the dynamic fluctuations of the brain [22]. As such, assessing dynamic changes rooted in the rTPJ might allow to deepen the understanding of FC alterations to underlying network alterations and fluctuations, and could provide further insights into the presently varying results in the literature.

On the other hand, FND is characterized by diverse phenotypes and studies including a heterogeneous patient cohort might prevent clear results. Evidence for common features [23] and shared pathophysiology [5] across subtypes have recently been opposed by highlighted phenotype diversity of clinical characteristics (e.g., symptom onset, gender distribution) [24] including neuropsychiatric features [25] or biological mechanisms underpinning different symptom manifestations [26]. A division based on symptom phenotype was proposed in Mueller *et al*. [27], which applied different network centrality measures in resting-state fMRI and found distinct pathophysiological mechanisms for patients with pronounced functional weakness compared to patients with motor symptoms (tremor, myoclonus, dystonia, gait disorder), particularly a stronger network centrality of the left TPJ and precuneus.

In regard of the above delineated role of the rTPJ in FND, we aimed to (1) evaluate functional alterations in the TPJ network between FND patients and age- and sex-matched healthy controls using a seed-based co-activation pattern-based approach and to (2) identify motor subgroup characteristics in patients as previously proposed by Mueller et al. [27]. By focusing on the rTPJ as a seed, our study addresses its heterogeneity in the literature and seeks to clarify its role in the *dynamic* network alterations associated with FND, which remain elusive in previous *static* approaches.

## Materials and methods

### Participants

This study selected 62 patients with functional motor symptoms from a mixed FND cohort along with 58 HC comparable in age and sex from a previously published dataset [28–30]. The Ethic Committee of Canton of Bern, Switzerland approved the study (2017-00997; SNCTP000002289), which was conducted according to the Declaration of Helsinki. Subjects provided written informed consent. Severity of functional motor symptoms were assessed using the Simplified Version of the Psychogenic Movement Disorder Rating Scale (S-FMDRS) [31]. Concomitant psychotropic medication was recorded and dichotomized (intake: yes/no). To control for anxiety and depression, both groups completed the Beck Depression Inventory (BDI) [32] and the State-Trait Anxiety Inventory (STAI) [33]. We divided the FND patients into two subgroups based on the phenotypic nature of their functional motor symptoms according to Mueller et al. [27]. Thereafter, patients were stratified into functional weakness (FW) or abnormal movements including tremor, dystonia, myoclonus and/or gait disorder (no-FW). Patients who presented with functional weakness and concomitant abnormal movements were classified as FW.

### Neuroimaging acquisition and pre-processing

A 3 Tesla scanner (Magnetom Prisma, Siemens, Germany) was used to record resting-state functional and structural MRI data (Supplementary Material for MRI sequence and pre-processing). Participants’ heads were stabilized using foam pads and resting-state fMRI data were subsequently evaluated for excessive translation and rotation in the x-, y-, z-direction with the framewise displacement (FD) criterion [34] at a threshold of FD > 0.5 mm. Subjects in which more than 30% of volumes showed motion exceeding this threshold were excluded from further analysis. For the subsequent analysis, temporal z-scoring was performed on voxel-wise time courses, and individual frames were scrubbed at 0.5 mm. The analyses were restricted to grey-matter voxels only.

### Functional network dynamics

To characterize the dynamic rTPJ network, we analysed its spatial and temporal characteristics, using a seed-based co-activation pattern (CAP) analysis [35, 36] according to Weber et al. [30] (detailed in Supplementary Material). The rTPJ seed was defined as a sphere of 10 mm centred at Montreal Neurological Institute (MNI) coordinates [62 −34 30] based on a previous study that identified hypoactivity in that localisation in FND patients compared to HC during a visuomotor task inducing reduced agency [13]. To probe the specificity of the rTPJ in the pathology of FND, we exploratorily investigated the functional network dynamics of the lTPJ (MNI [−62 –34 30], Supplementary Material). Building CAPs allows the characterization of dynamic connectivity patterns from this seed to the rest of the brain, capturing transient co-activation and co-deactivation events that complement static FC measures. Increased temporal measures indicate more co-activation with the seed, suggesting increased coupling. Correspondingly, reduced temporal measures of co-activation patterns indicate less frequent co-activation with the seed, reflecting decreased coupling. In co-deactivation patterns, increased temporal measures represent stronger transient anti-correlations with the seed, i.e., reduced coupling, while decreased temporal measures of co-deactivation patterns indicate weaker anti-correlations, reflecting transiently increased coupling [37]. Unlike *static* FC, CAP analyses highlight dynamic connectivity changes that can reveal patterns not apparent in traditional FC approaches.

The CAP analysis included the following steps: 1) a selection of time-points (functional volumes) in which the rTPJ was highly active, i.e., corresponding to high-amplitude events at a threshold of 0.84 SD (80^th^ percentile) [38], 2) a dimensionality reduction of the data using a principal component analysis (PCA) step, 3) identification of optimal number of clusters *K* using a consensus clustering approach on the dimensionality reduced functional volumes for which the proportion of ambiguously clustered pairs (*PAC*) was evaluated to assess the stability of the individual cluster sizes [39] (the stability measure, defined as 1-*PAC*, and associated consensus matrices can be found in the Supplementary Material), 4) clustering into four CAPs based on the *PAC* and consensus matrices and back-reconstruction of individual CAPs by multiplying the principal component scores with the transposed eigenvectors and adding the mean.

#### Comparison of CAPs between HC and FND

To address our first aim, we defined transient rTPJ co-activation patterns derived from HC. Only the functional volumes from HC were used during the consensus clustering and subsequent clustering into the four individual CAPs, representing the dynamic rTPJ network of HC. To evaluate how the dynamics of the rTPJ network of FND patients deviates from those in HC, the individual volumes of FND patients were assigned to the most similar CAP derived from HC using a matching process. During the matching process, each volume of the FND patients was assigned to the CAP to which it was the most spatially correlated – provided that this correlation exceeded the 5^th^ percentile of the distribution of spatial correlations of the functional volumes of HC belonging to that best matching CAP. In order to objectively quantify the networks within each identified CAP, we computed the overlap (in %) between the significant voxels of each CAP with the Yeo atlas [40]. The Yeo atlas contains various resting-state networks in line with their different functional network organisation and is therefore suitable to study rTPJ network dynamics. Seed voxels were excluded. To evaluate how FND patients deviate from HC with respect to dynamic functional alterations, we calculated the number of transitions (“Entries”; i.e., how many times a subject entered a specific CAP), and the average duration of the individual CAPs (“Duration”; i.e., the average number of sequential volumes assigned to a CAP multiplied by the TR). We included the number of excluded fMRI frames (derived from FD), number of selected volumes, age, sex, psychotropic medication (dichotomous), depression (BDI) and state anxiety (STAI) scores as covariates to the analyses and corrected the analyses for multiple comparisons using False Discovery Rate (FDR). Lastly, we performed exploratory correlation analyses between the temporal measures and clinical variables, including disease severity (S-FMDRS), duration of illness, BDI, STAI-S, and STAI-T, to provide additional context regarding the clinical relevance of the dynamic connectivity findings. All analyses were repeated with the second most stable cluster number (Supplementary Material). For comparison, a static functional connectivity analyses with the rTPJ as seed region has been performed in the Supplementary Material.

#### Comparison of CAPs between FW and no-FW

To address our second aim, we evaluated the rTPJ network dynamics within FND subtypes based on previous results [27] which indicated that network dynamics might be altered differently across symptom types. On these grounds, we computed rTPJ co-(de)activation patterns derived from FND patients only to identify FND-specific dynamic alterations between subtypes. Thus, we repeated the analyses (without the matching process), including the computation of CAPs and subsequent statistical analysis of temporal characteristics between FND patients with FW and those without FW (no-FW). To assess the spatial similarity between CAPs derived from HC and those from patients with FND, we calculated the percentage overlap of voxels for each CAP. Overlap was determined by comparing the spatial distribution of each CAP from the two groups, expressed as the proportion of voxels shared between the corresponding CAPs.

## Results

### Demographic and clinical characteristics

As four patients were excluded due to too high motion artefacts during fMRI, this study included 58 patients with functional motor symptoms to which we matched 58 healthy controls comparable in age and sex. The cohorts significantly differed in depression and anxiety scores, with higher levels in patients. Demographic and clinical data are presented in Table 1.

**Table 1:** Demographic and clinical characteristics of the sample.

	FND (*N* = 58)	HC (*N* = 58)	Statistics
Age, mean (SD), years, [range]	35.5 (12.8), [17–69]	34.1 (12.0), [18–62]	*Z* = −0.49, *P* = 0.62
Sex (females/males)	43/15	45/13	*X*^*2*^ (1, 116) = 0.01, *P* = 0.92
Disease severity (S-FMDRS, median, quantile)	6.5 [2 −13.0]	*NA*	
Duration of illness (in months, SD)	54.71 (66.5)	*NA*	
Symptom type^a^	38 sensorimotor 21 gait disorder 14 tremor 10 myoclonus 6 dystonia 1 speech disorder 1 functional vision loss 2 functional hearing loss	*NA*	
Symptom subtype (FW vs. no-FW)	29/29	*NA*	
Psychotropic medication (yes/no)	28/30	0/58	*Not applicable*
BDI score, mean (SD)	14.4 (10.0)	4.59 (6.3)	*Z* = −6.8, *P* < 0.0001 ***
STAI-S score, mean (SD)	37.2 (10.9)	32.1 (7.7)	*t(*89.3) = 3.64, *P* = 0.0004 ***
STAI-T score, mean (SD)	45.5 (13.0)	33.9 (7.1)	*t*(87.5) = 6.8, *P* < 0.0001 ****
Number of excluded frames, mean (SD)	20 (34.6)	4.59 (16.6)	*Z* = −4.52, *P* < 0.0001 ****
Number of frames with rTPJ activation, mean (SD)	55.8 (10.8)	61.2 (6.7)	*t*(95.7) = −3.3, *P* = 0.001 **

*BDI* Beck’s depression inventory, *FND* functional neurological disorders, *FW* functional weakness, *HC* healthy controls, *no-FW* no functional weakness (abnormal movements), *S-FMDRS* simplified version of the psychogenic movement disorder rating scale, *STAI* state-trait anxiety inventory, *SD* standard deviation, *ns* not significant, *rTPJ* right temporoparietal junction, *NA* not applicable.

Significance code: ****P < 0.0001, ***P < 0.001, **P < 0.01.

^a^Patients can present with several symptom types.

### rTPJ brain network dynamics in healthy controls

**Fig. 1:**
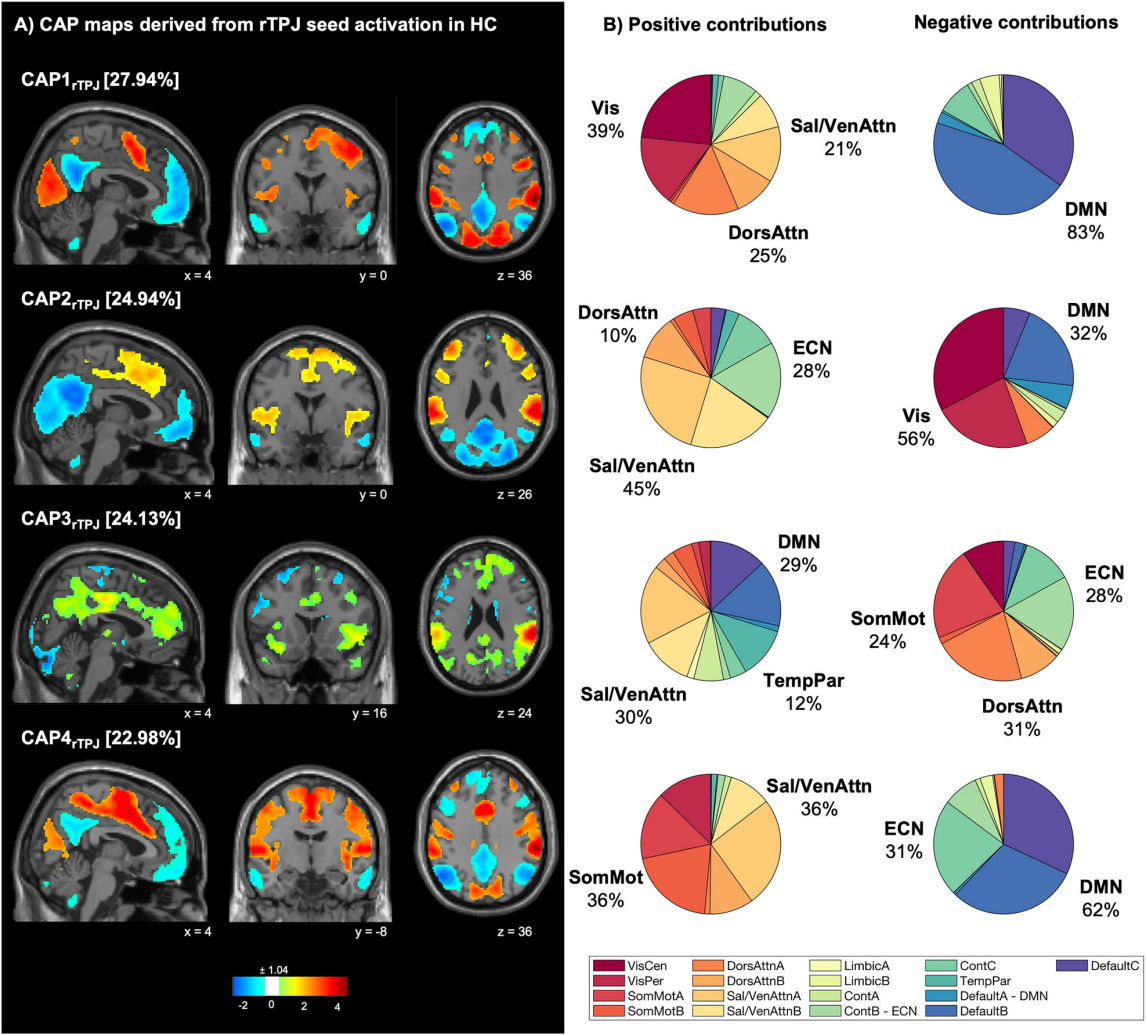
Co-activation pattern (CAP) maps based on rTPJ seed activation derived from healthy controls (HC). **A** Four CAPs were detected. CAPs were z-scored and only the 15% most positive and 15% most negative contributions are represented in colour (z = ± 1.04), with red representing positive contributions and blue negative contributions. Locations are displayed in Montreal Neurological Institute (MNI) standard space coordinates. **B** Pie charts illustrating the percentage of positive and negative contributions within the 17 resting-state networks according to the convention of Yeo [40]. Seed voxels were removed. rTPJ right temporo-parietal junction, HC healthy controls, Cont executive control, Default default mode DorsAttn dorsal attention, Sal/VenAttn salience/ventral attention, SomMot somatomotor, TempPar temporoparietal, VisCen central visual, VisPer peripheral visual.

Frames with rTPJ activation, defined as exceeding the 80^th^ percentile of the seed time course, were less frequent in FND (mean = 55.8, SD = 10.8) compared to HC (mean = 61.2, SD = 6.7; *t*(95.7) = −3.3, *P* = 0.001). We identified four CAPs (“*states*”) corresponding to rTPJ co-activations in the control cohort. The first CAP (CAP1_rTPJ_; CAP1-VisualAttention) exhibited an rTPJ co-activation with the visual network (Vis [total Yeo overlap: 39%]), the dorsal attention network (DorsAttn [25%]) and the salience/ventral attention network (Sal [21%]), including regions such as the supplementary motor area (SMA), and the middle cingulate cortex (MCC); and a co-deactivation with the default mode network (DMN [83%]), including the posterior cingulate cortex (PCC), the precuneus and the orbitofrontal cortex (OFC). The second CAP (CAP2_rTPJ_; CAP2-SalienceAttention) represented an activation pattern with co-activation of the Sal [45%] and executive control network (ECN [28%]), and regions including the left TPJ, the insula, the MCC and the SMA; and a co-deactivation with the Vis [56%] and DMN [32%], including the precuneus, the OFC and lingual gyrus. The third CAP (CAP3_rTPJ_; CAP3-DMN-TemporoParietal) denoted an rTPJ activation pattern with co-activation of the Sal [30%], DMN [29%], and temporoparietal network (TempPar [12%]), including regions such as the thalamus, the left TPJ, the MCC, the SMA, the superior frontal gyrus and the precuneus; as well as co-deactivation of the somatomotor network (SomMot [24%]), the DorsAttn [31%] and the ECN [28%], and regions such as the caudate, the inferior frontal gyrus, the fusiform gyrus and the postcentral gyrus. The last CAP (CAP4_rTPJ_; CAP4-SomatoMotor) denoted an activation pattern with co-activation of the SomMot [36%] and Sal [36%], including the SMA, the ACC, the supramarginal gyrus, the frontal middle gyrus and the insula; and a co-deactivation with the DMN [62%] and ECN [31%], including the PCC, the MCC, the precuneus, the inferior frontal gyrus, the middle temporal gyrus and the OFC, Fig. 1.

**Fig. 2:**
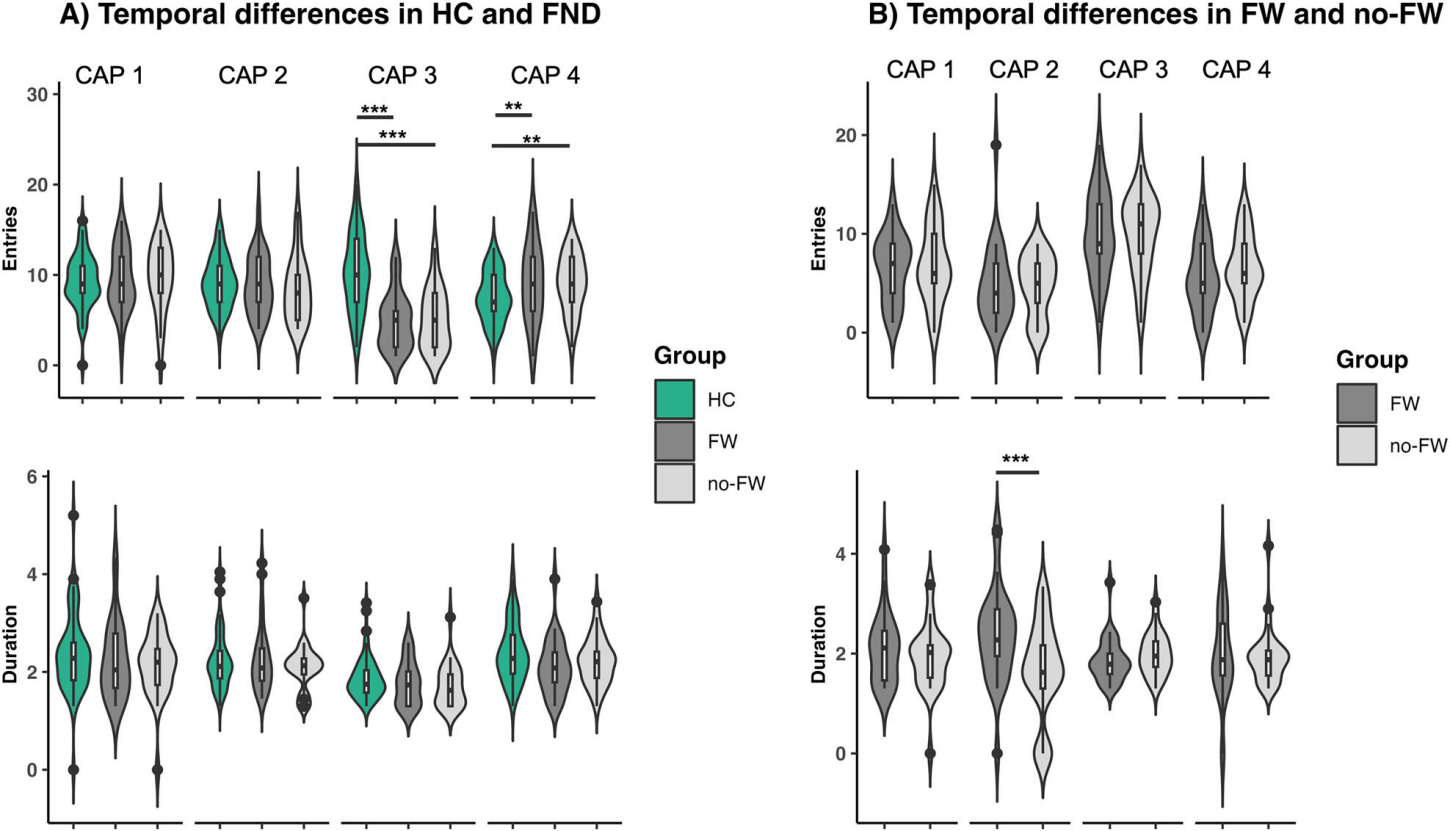
Co-activation pattern (CAP) temporal measures. For CAPs derived from healthy controls compared to FND patients (**A**) and CAPs derived from FND patients compared between different symptom types (**B**) numbers of entries (top), and duration (bottom) are displayed. Violin plots: visualize the distribution of the data. Boxplots: horizontal lines represent group median; box represents interquartile range, and vertical line represents 1.5-times interquartile range. Asterisks indicate statistical significance for data adjusted for covariates (i.e., number of excluded fMRI frames [derived from FD], number of selected volumes, age, sex, psychotropic medication [dichotomous], depression [BDI] and anxiety [STAI] scores) and corrected for multiple comparisons using FDR with *p < 0.05, **p < 0.01, ***p < 0.001. BDI Beck’s Depression Inventory, STAI State-Trait Anxiety Inventory.

27.94% of functional volumes were assigned to CAP1-VisualAttention, 24.94% to CAP2-SalienceAttention, 24.13% to CAP3-DMN-TemporoParietal, and 22.98% to CAP4-SomatoMotor. Group comparisons of temporal characteristics revealed a significant group difference in entries for factors *state* (*F*(3,445) = 6.8, *P* = 0.0002), as well as in duration for the factors *state* (*F*(3445) = 17.3, *P* < 0.0001). Post-hoc multiple comparisons revealed that FW patients entered CAP3-DMN-TemporoParietal less frequently than HC (*P*_*FDR*_ < 0.0001) and entered CAP4-SomatoMotor more often than HC (*P*_*FDR*_ = 0.009). Likewise, no-FW patients entered CAP3-DMN-TemporoParietal less frequently than HC (*P*_*FDR*_ < 0.0001) and entered CAP4-SomatoMotor more often than HC (*P*_*FDR*_ = 0.009). No significant differences were found regarding duration, Fig. 2A. No significant correlations were found between the temporal measures and the clinical variables.

### rTPJ brain network dynamics in motor FND subtypes

**Fig. 3:**
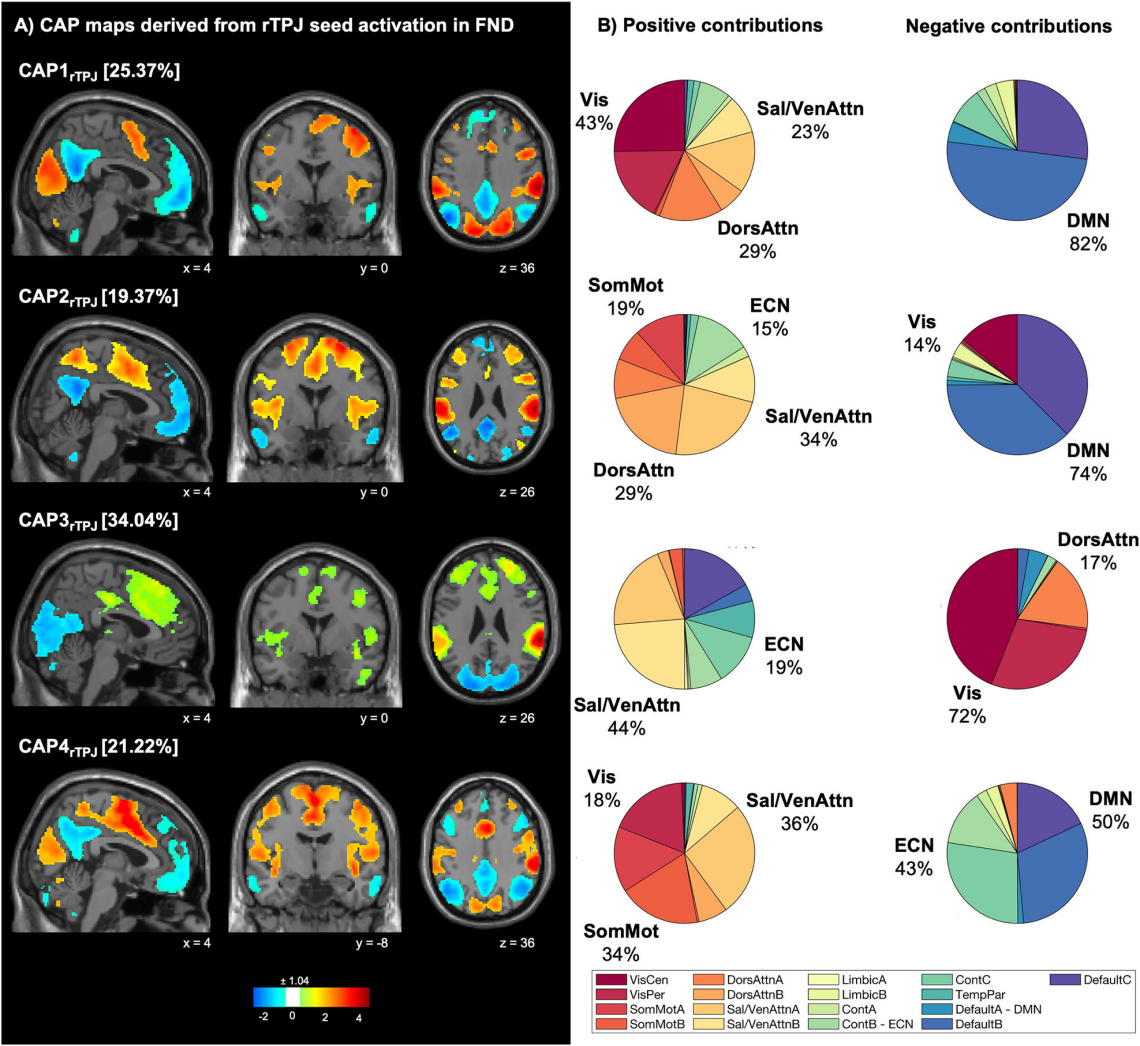
Co-activation pattern (CAP) maps based on rTPJ seed activation derived from FND patients. **A** Four CAPs were detected. CAPs were z-scored and only the 15% most positive and 15% most negative contributions are represented in colour (z = ± 1.04), with red representing positive contributions and blue negative contributions. Locations are displayed in Montreal Neurological Institute (MNI) standard space coordinates. **B** Pie charts illustrating the percentage of positive and negative contributions within the 17 resting-state networks according to the convention of Yeo [40]. Seed voxels were removed. rTPJ right temporo-parietal junction, FND motor functional neurological disorders, FW functional weakness, no-FW no functional weakness (abnormal movements), Cont executive control, Default default mode, DorsAttn dorsal attention, Sal/VenAttn salience/ventral attention, SomMot somatomotor, TempPar temporoparietal, VisCen central visual, VisPer peripheral visual.

Four co-activation patterns were identified corresponding to rTPJ co-activation in FND patients. The first CAP (CAP1_rTPJ_; CAP1-VisualAttention) was characterized by an rTPJ activation pattern with co-activation of the DorsAttn [total Yeo overlap: 29%], Sal [23%] and Vis [43%], covering regions such as the SMA, the MCC, the cuneus, the supramarginal gyrus and the lingual gyrus; as well as a co-deactivation of the DMN [82%], including the middle temporal gyrus, the OFC, the precuneus and the PCC. The second CAP (CAP2_rTPJ_; CAP2-SalienceAttention) showed an activation pattern with co-activation of the SomMot [19%], DorsAttn [29%], Sal [34%] and ECN [15%], including the precuneus, the SMA, the MCC, frontal middle gyrus and the insula; with a co-deactivation of the DMN [74%], including the precuneus, the PCC and the OFC. The third CAP (CAP3_rTPJ_; CAP3-DMN-ECN) showed an activation pattern with co-activation of the Sal [44%], and ECN [19%], and regions including the left TPJ, the inferior frontal gyrus, the inferior temporal gyrus, the SMA, the precentral gyrus and the insula; and a co-deactivation with the Vis [72%] and DorsAttn [17%], including the pre-SMA, the inferior parietal gyrus and the middle occipital gyrus. The last CAP (CAP4_rTPJ_; CAP4-SomatoMotor) depicted an rTPJ activation pattern with co-activation of the SomMot [34%] and Sal [36%], covering the SMA, the PCC, the MCC, the cuneus and the insula; as well as co-deactivation of the DMN [50%] and ECN [43%], including the angular gyrus, the middle frontal gyrus, the precuneus and the OFC, Fig. 3. 25.37% of the volumes were assigned to CAP1-VisualAttention, 19.37% to CAP2-SalienceAttention, 34.04% to CAP3-DMN-ECN, and 21.22% to CAP4-SomatoMotor. The CAPs derived from FND showed high voxel overlap with those from HC: CAP1_rTPJ_ (96%), CAP2_rTPJ_ (86%), CAP3_rTPJ_ (84%), and CAP4_rTPJ_ (94%).

Subtype comparisons (FW vs. no-FW) of temporal characteristics, revealed a significant group difference in entries for factors *state* (*F*(3,445) = 6.8, *P* = 0.0002), as well as in duration for the factors *state* (*F*(3,445) = 17.3, *P* < 0.0001). Post-hoc multiple comparisons identified that CAP2-SalienceAttention was of longer duration in FW patients (*N* = 29) compared to no-FW patients (*N* = 29; *P*_*FDR*_ = 0.0006). No significant differences were found regarding entries, Fig. 2B. Specific correlations with clinical variables were observed in the FW group, where entries into CAP3 showed a trend-level correlation with duration of illness (*r* = 0.36, *P* = 0.058), while entries into CAP2 significantly correlated with STAI-S scores (*r* = −0.42, *P* = 0.02). No significant correlations were identified in the no-FW group.

## Discussion

Using a co-activation-based approach, our data showed that dynamic connectivity was different between FND patients and HC in terms of entry rates into two brain states: 1) a co-activation of the right TPJ with the DMN and temporo-parietal network (CAP3 DMN-TemporoParietal; with patients entering this state less often) and 2) a co-activation with the somatomotor network (CAP4-SomatoMotor; with patients entering this state more often). Our subgroup analyses revealed that patients with symptoms of weakness (FW) stayed for a longer duration in a brain state characterized by co-activation of the right TPJ and dorsal/ventral attentional networks (CAP2-SalienceAttention) and somatomotor regions compared to patients with other motor FND.

### Altered network coupling distinguishes FND patients from healthy controls

Comparing characteristics in temporal connectivity from the rTPJ to the rest of the brain between FND patients and HC, we first identified less entries for patients in a network engaging the DMN and temporo-parietal regions (Fig. 1, CAP3-DMN-TemporoParietal), concomitant with deactivation of the executive network (Fig. 1, CAP3-DMN-TemporoParietal). Temporo-parietal regions and the SMA included in CAP3-DMN-TemporoParietal are of particular interest, as those areas have been associated with the formation of the SoA [41]. The findings of reduced entries in this state in patients could thus suggest that a pathophysiological mechanism in FND may involve less ability to turn down the executive control and enter a state where the network underpinning the SoA is active together with self-referential areas of the DMN, such as the precuneus, which is important in self-monitoring [27].

Second, patients entered more often than controls a brain state characterized by rTPJ co-activation with the somatomotor network (Fig. 1, CAP4-SomatoMotor), involving the SMA, concomitant with an anti-coupling with the DMN and executive control network (Fig. 1, CAP4-SomatoMotor). This suggests that a pathophysiological mechanism in FND may involve abnormally high activity of the motor networks at rest compared to controls.

#### The role of the salience network and temporo-parietal regions

The first interesting pattern is the altered coupling behaviour of the rTPJ with the salience network and temporo-parietal areas with concomitant DMN coupling and anti-coupling of the ECN. Noteworthy, the connectivity between the salience network and the rTPJ has previously been implicated in stimulus-driven attention and detection of relevant internal and external cues [42]. The salience network plays a central role when switching between large-scale networks [43], such as for example when directing the attention to a salient stimulus by modulating the engagement of the ECN and the simultaneous disengagement of the DMN [43]. Furthermore, the DMN and ECN – usually anti-correlated networks – represent competing networks with regards to external versus internal processing of stimuli [44, 45], for which an increase in ECN-Sal activity is usually observed during stimulus-driven processes [46]. The herein reported alterations in brain states might indicate difficulties in flexibility switching between brain states in FND.

#### The role of the somatomotor network

The second interesting finding is the increased interaction between the rTPJ and sensorimotor regions in FND patients. Only one other study [19] assessed resting-state FC derived from the rTPJ as seed region in 35 motor FND patients and compared the data to 35 age- and sex-matched controls. In this study, FND (mixed motor symptoms, 31% presenting with functional weakness) showed decreased FC between rTPJ and right sensorimotor cortex, cerebellar vermis, bilateral SMA and right insula, which the authors subsumed as impaired feed-forward signals and altered sensory feedback from sensorimotor integration areas that might alter patients’ SoA. It is of note, that this previous work was based on *static* FC measures, which might explain the apparent inverse directionality of our results (our data showing increased coupling), as our method brings additional information on temporal fluctuation of brain activity. In fact, both studies point towards abnormal connectivity between the rTPJ and the somatomotor network. Findings from Maurer, et al. [19] demonstrate a decoupling of the rTPJ with the somatomotor network, while our findings demonstrate that patients switch more often to a state where the rTPJ is coupled with the somatomotor network and decoupled with the executive and DMN networks. This supports the hypothesis of impaired executive control or feedforward signals on motor control in FND. With regards to motor symptoms, altered coupling between the default mode and sensorimotor networks has been associated to gait impairments in neurodegenerative [47] and normal pressure hydrocephalus (NPH) [37] patients, where altered interactions between the DMN, the ECN and the salience network were significantly associated with gait symptomatology. Moreover, gait disturbances were found to normalize together with these altered brain dynamics after cerebrospinal fluid tap test, indicating functional plasticity mechanisms that directly affect gait performance. These previous findings emphasize how dynamic brain network interactions can add to motor performance and support the herein reported results. Therefore, abnormal dynamic network interactions in FND might contribute to the observed motor symptomatology.

Likewise, using *dynamic* functional measures with the posterior cingulate cortex as a seeding region, altered network dynamics have been reported in patients with functional hyperkinetic movement disorders in which patients transitioned more often between states associated with the DMN [48] as compared to HC, which has been interpreted as a potential compensatory mechanism of altered self-referential processes as previously observed in FND [49, 50].

By complementing our dynamic FC approach with contemporary static FC analyses (Supplementary Material), we did not observe any significant between-group differences. To further evaluate the specificity of the rTPJ in the pathophysiology of FND, we exploratorily replicated the analyses with the lTPJ as a seed (Supplementary Material). Given those results and previously reported lTPJ contribution in FND [27], we postulate the lTPJ being partially involved in but extricable from rTPJ functionalities relevant to FND and recommend further research to unravel the specific roles of TPJ laterality in FND.

### Different coupling duration in patients with functional weakness compared to abnormal movements

Dividing the patient cohort into symptom phenotypes allows investigating potential phenotype-specific characteristics. Our data demonstrated a difference between patients with functional weakness (FW) compared to patients with other types of abnormal movements (no-FW) with longer duration of CAP2 in FW patients. This means that patients with FW display increased coupling duration between rTPJ and the salience and attention networks as well as the somatomotor network and executive network. A concomitantly reduced coupling duration in FW patients between the rTPJ and the DMN was found compared to no-FW patients (Fig. 3, CAP2-SalienceAttention). This suggests a mechanism of abnormally enhanced coupling between the rTPJ involved in the SoA and somatomotor networks that would be more pronounced in FW. Previous static connectivity analyses from Maurer, et al. [19] performed on a cohort of mixed FND with predominant abnormal movements and only a third of FW, revealed a decreased coupling between the rTPJ and the somatomotor areas. This evidence together with our data could suggest that patients with no-FW have decreased coupling whereas patients with FW have increased coupling between rTPJ and other motor network nodes.

Another recent study specifically looking at differences between FW and no-FW symptoms in resting state fMRI [27] identified increased network centrality in left TPJ and precuneus (related to DMN) in FW relative to no-FW and HC. Comparing FW to HC, a reduced brain network centrality in the insula and the SMA was found. This hyperconnectivity in the TPJ correlated with symptom severity in FW, which might represent a potential biomarker for this subtype of FND. The left TPJ seems involved in attention (non-spatial, motor, monitoring of attention) and is (together with rTPJ) linked to predictions within a Bayesian Inference Model, assumingly updating and adjusting top-down predictions. As we selected the TPJ in the right hemisphere, which has been consistently associated to social cognition and authorship attribution, we can extend the neuropathophysiological differentiation between subtypes of FW and no-FW in regard to self-referential processes including the SoA or attention as both are described as relevant constructs in the multi-network model of FND [2].

As we observed higher DMN anti-coupling duration in FW (Fig. 3, CAP2-SalienceAttention), the role of the DMN was highlighted recently in a sample of seven FND patients with stroke-like symptoms reflected in unilateral paresis and hypoesthesia [20]. Compared to 15 controls, the authors identified an increased FC strength within the DMN network during RS-fMRI, which they supposed to represent elevated self-referential processing, that eventually stands in interference with motor activity and thus contributes to the phenomenology of weakness. Our analyses revealed a subtype difference with regards to a reduced coupling duration in CAP2-SalienceAttention involving predominantly the DMN in FW patients compared to no-FW. As such, looking at the subgroup of FW that display similar phenomenology as the subjects in Monsa, et al. [20], we found a longer duration of DMN co-deactivation in FW compared to no-FW, sustaining this previous observation. Likewise, a longer duration of prefrontal (laying within the DMN) and visual regions with enhanced salience network activity has been suggested to be reflected as a decrease in cognitive control, consequently with a heightened focus on self-reflective information [51].

Previously, enhanced ventromedial prefrontal cortex activity – as part of the DMN – has been found in patients which was attributed to enhanced self-monitoring that negatively interacts with motor pathways, i.e., reflecting a dysfunction in *motor initiation* in FND [50, 52]. This dysfunction in motor initiation was extended to an impaired *awareness* of voluntary motor initiation [6, 7], associated to increased activity between the prefrontal cortex and the SMA [7]. In line with this, it could be shown that voluntary, straight movements of functional tremor patients worsened when attention was explicitly shifted towards visual feedback but improved with a shift of the attention to different aspects of the movement, e.g., increasing its velocity [53]. Thus, altered network connectivity might point towards an impairment upstream of motor intention and action selection, and is thought to be involved in the inhibition of already ongoing motor programs [54], which aligns with our findings on altered entry rate and CAP duration - for example by constantly interfering and updating ongoing motor programs (no-FW patients) or by remaining for too long in a certain state, thus not updating the ongoing program (FW patients). Therefore, altered salient and attentional processes in interaction with the DMN and somatomotor network might interfere with the implicit (“automatic”) execution of a motor program by replacing it with an explicit *control of movement*, which is then normally less smooth and less well performed [55]. As a consequence, the attribution of self-authorship gets distorted, with the rTPJ as comparator region being inadequately matching original intention and sensory feedback which is leading to the perception of involuntariness. The neural correlates of the involuntariness during functional symptoms were investigated earlier, in a study during which brain activity of FND patients was examined in the very same moment they experienced their functional tremor [14]. As compared to the activation during voluntarily mimicking their symptoms, a hypoactivation in the right TPJ was found in patients during the involuntarily experienced symptoms. These findings connect the herein reported results on altered rTPJ coactivation pattern with the DMN, the salience network, the ECN and SomMot within the framework of an impaired awareness of motor intention and control of movement in FND patients. In summary, these findings emphasize that functional symptoms might arise as a pathology of the attentional/salience networks, the DMN and ECN rather than – or in combination with - a dysfunction of the motor system.

### Limitations

Regarding the selection of a seed, we defined the rTPJ as a rigid sphere around literature based MNI-coordinates. However, the rTPJ as neural correlate of SoA has been reported with slightly different localizations [56] and our seed might therefore not optimally cover each participant’s functional equivalent. Furthermore, as caveat of resting-state based studies, the translation of FC at rest to brain processes during interactions or task performance (e.g., attention, agency attribution, control of movement) remains speculative. On a technical level, seed-based co-activation pattern analyses bear the risk that co-activation with the seed might occur at chance level without direct functional importance [36] and might be susceptible to noise. Furthermore, the selection of the optimal cluster size based on consensus might be common practice but could result in the selection of a less optimal cluster. We counter steered this limitation by examining also other cluster sizes, which helps understanding how the different clusters were built. Also, the implementation of a dimensionality reduction step might cause the loss of meaningful signal. In our previous work [30], we could show that the PCA step provides a valuable trade-off between high computational load and potential loss of weaker networks. There is no clear consensus on the physiological interpretation of altered temporal characteristics of co-activation patterns in the brain. Our findings hint towards a potential association of symptom duration and state anxiety in FND patients with FW, but we emphasize that further studies need to confirm the clinical relevance of dynamic FC measures regarding the symptom development and clinical features in FND. Regarding clinical phenotypes, although we focused on patients with motor symptoms only, the symptom types differ across the FND population, which affects the generalizability of the results. Additionally, we did not perform a systematic psychiatric evaluation and thus, cannot exclude that the results were driven by psychiatric comorbidities commonly observed in FND [57]. Similarly, we did not exclude patients who were currently under psychotropic medication, which might influence functional alterations in the brain. However, we corrected our analyses for a potential effect arising from depression, anxiety, or intake of psychotropic medication. Lastly, we acknowledge that beyond symptom type variability, other factors may contribute to the heterogeneity in functional and structural neuroimaging results in FND (e.g., symptom severity, duration of illness or comorbid diagnoses, MR-sequence parameters, control group selection). We applied a previously reported symptom-based stratification [27] and thoroughly reported sample characteristics and methods to enhance comparability, aiming for a more comprehensive understanding of FND brain alterations.

## Conclusion

Using a co-activation-based approach, we identified differences in the rTPJ co-(de)activation patterns associated with the ECN, the DMN and the somatomotor network in patients with FND compared to HC. Within the framework of a subgroup analysis, we identified alterations in the rTPJ co-(de)activation pattern duration associated with the attention networks (dorsal and ventral/salience) and the DMN comparing patients with FW to those with no-FW. The underlying disbalance between the attention networks, the DMN, the ECN, and the somatomotor network with the rTPJ might reflect difficulties in attention relocation and altered self-referential processes in FND which negatively interact with the proper planning and execution of motor programs, with the rTPJ as a potential key node for multimodal integration of information.

## Supplementary information

Supplementary Material

## Data Availability

The data are not publicly available but can be shared upon request. The code is available under https://github.com/webersamantha/PCA_CAP_SW.
