# Supplementary material for: Altered brain network dynamics in motor functional neurological disorders: the role of the right temporo-parietal junction
Source: Transl Psychiatry. 2025 May 15;15:167. doi: 10.1038/s41398-025-03385-5 (PMC12081630; doi:10.1038/s41398-025-03385-5)
Supplement: Supplementary file 1 — Supplementary Material [file 41398_2025_3385_MOESM1_ESM.pdf]

Supplementary Material

Altered brain network dynamics in motor functional neurological disorders: The role of the right temporo-parietal junction.

Samantha Weber[1,2,3,4*], Janine Bühler[1,4,5,6*], Thomas A.W. Bolton[7], Selma Aybek[1,6]

[1] Department of Neurology, Inselspital, Bern University Hospital, University of Bern, Bern, Switzerland.

[2] University Hospital of Psychiatry Zurich, Department of Adult Psychiatry and Psychotherapy, 8032 Zurich, Switzerland.

[3] Faculty of Medicine, University of Zurich (UZH), 8006 Zürich, Switzerland.

[4] Translational Imaging Center (TIC), Swiss Institute for Translational and Entrepreneurial Medicine, 3010 Bern, Switzerland.

[5] Danish Research Centre for Magnetic Resonance, Department of Radiology and Nuclear Medicine, Copenhagen University Hospital - Amager and Hvidovre, Copenhagen, Denmark.

[6] Faculty of Science and Medicine, University of Fribourg, Fribourg, Switzerland.

[7] Connectomics Laboratory, Department of Radiology, Centre Hospitalier Universitaire Vaudois (CHUV), 1011 Lausanne, Switzerland.

[*] Samantha Weber and Janine Bühler contributed equally to this work.

Neuroimaging data acquisition and pre-processing

For anatomical imaging, a sagittal-oriented T1-weighted 3D-MPRAGE sequence (TR = 2330 ms, TE = 3.03 ms, TI = 1100 ms, matrix 256 × 256, FOV 256 mm × 256 mm, flip angle 8°, resolution 1 mm^3 isotropic, TA = 5:27 min) was acquired for all subjects.[1] Functional imaging data were acquired using a whole-brain interleaved multi-slice BOLD echo-planar-imaging (EPI) sequence (TR = 1300 ms; TE = 37 ms, flip angle = 52°, FOV = 230 mm, voxel size = 2.2 mm^3 isotropic, TA = 6:39 min, for a total of 300 functional volumes). Imaging data were pre-processed using SPM12 (https://www.fil.ion.ucl.ac.uk/spm/software/spm12/) in MATLAB (R2017b, MathWork Inc., Natick, USA). Functional volumes were first realigned and co-registered to the structural T1 volume. They were then detrended and covariates of no interest were regressed out (including constant, linear, and quadratic trends, average white matter/cerebrospinal fluid time courses, motion artefacts, and global signal). Functional data were then filtered using a high-pass filter at 0.01 Hz. Lastly, functional volumes were warped into MNI standard space and smoothed using a spatial Gaussian kernel of 5 mm full width at half maximum.

Static Functional Connectivity of the Temporoparietal Junction

Whole-brain resting-state functional connectivity (FC) was calculated according to standard procedure.[2–5] As such, the functional images were parcellated into the 92 of which 90 originated from cortical and subcortical regions of interest according to the automatic anatomic labelling atlas (AAL) and the two remaining regions representing the right and left temporoparietal junction (TPJ). The rTPJ and lTPJ were created using a 10mm sphere mask around the following coordinates: rTPJ [62 -34 30] and lTPJ [-62 -34 30]. The region-averaged time courses were extracted, and TPJ-to-whole brain FC was computed using Pearson's correlation coefficient between the time series of the two seeds with each of the AAL regions. The correlation coefficients were further z-scored using Fisher z transformation. Significant differences in functional connectivity between FND patients ($N = 58$) and healthy controls (HC; $N = 58$) were assessed using two-tailed multiple t-tests at a significance threshold of $P < \alpha$, where alpha level (α) was set to 0.001.

Uncorrected for multiple comparisons, patients showed increased functional connectivity between the right TPJ and 1) the left superior frontal gyrus ($t(112) = -2.9$, $P = 0.004$), 2) the left medial orbital frontal gyrus ($t(101) = -2.6$, $P = 0.009$), 3) the left gyrus rectus ($t(102) = -3.5$, $P = 0.0008$), 4) the right lingual gyrus ($t(113) = -2.8$, $P = 0.005$), 5) the right inferior parietal cortex ($t(113) = -2.9$, $P = 0.003$), 6) the right calcarine gyrus ($t(110) = -3.1$, $P = 0.002$) and 7) the right middle temporal pole ($t(103) = -3.2$, $P = 0.002$), compared to HC. Moreover, patients showed decreased functional connectivity between the right TPJ and the right inferior frontal gyrus pars opercularis ($t(113) = 2.7$, $P = 0.007$). There were no significant differences in left TPJ connectivity between patients and controls. None of these results survived correction for multiple comparisons. Results uncorrected for multiple comparisons are visualized in Supplementary Figure 1, where increased FC in patients compared to controls is depicted in red and decreased FC in patients compared to controls is depicted in blue.

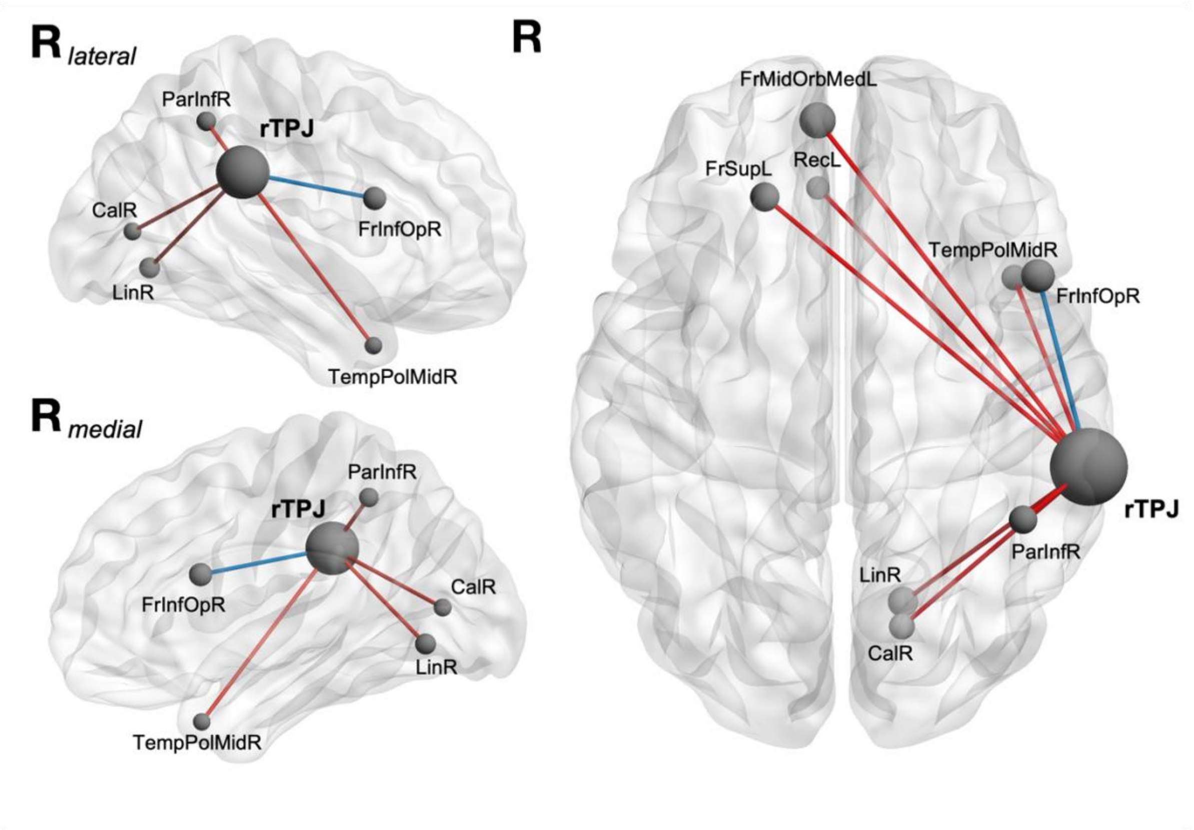

Supplementary Figure 1. Functional connectivity differences between FND patients and healthy controls – uncorrected for multiple comparisons. Right temporoparietal junction (rTPJ) to whole-brain resting-state functional connectivity (FC) was calculated by parcellating functional images into 92 regions of which 90 originated from cortical and subcortical regions based on the AAL atlas and the two remaining regions representing the right and left temporoparietal junction (TPJ). FC was computed using Pearson's correlation between region-averaged time courses and transformed using Fisher z-scores. Significant differences in functional connectivity between patients ($N = 58$) and controls ($N = 58$) were assessed using two-tailed multiple t-tests at a significance threshold of $P < \alpha$, where alpha level (α) was set to 0.001. Decreased functional connectivity in patients compared to healthy controls is depicted in blue, while increased functional connectivity is depicted in red. Abbreviations: CalR: right calcarine gyrus; FrInfOpR: right inferior frontal gyrus pars opercularis; FrMidOrbMedL: the left medial orbital frontal gyrus; FrSupL: left superior frontal gyrus; LinR: right lingual gyrus; ParInfR: right inferior parietal cortex, RecL: left gyrus rectus; rTPJ: right temporoparietal junction; TempPolMidR: right middle temporal pole

Co-Activation Pattern Analysis

To identify those time-points corresponding to high-amplitude events (activation) within our seed, we thresholded the time-series at 0.84 SD[6] which corresponds to the 80th percentile, representing high-amplitude blood oxygenation level-dependent signals.[7] This threshold was chosen to balance sensitivity and specificity, ensuring sufficient data coverage for robust analysis while focusing on significant activations. Furthermore, this approach has been validated in our previous work[8,9] and was observed to yield spatial patterns that were visually more distinct and interpretable. To identify the optimal number of clusters *K*, a consensus clustering approach was performed. Due to the high dimensionality of the data, and the consequential high computational load for clustering approaches, an additional principal component analysis (PCA) step was introduced to reduce the dimensionality of the data. Hence, we concatenated the data of each subject (of size *n* ´ *t*, where *n* is the number of grey matter voxels and *t* the number of timepoints selected per subject) into a data matrix *X* with dimensionality *n* ´ *T*, where *T* is the selected number of timepoints across all subjects and $T<<n$. The *X* matrix was then centred by subtracting the mean of each voxel. *X* further served as an input to PCA. The PCA projected data (scores) *W* (of dimension *T* ´ *T*) were used as input for consensus clustering. Based on the output of the consensus clusters, the cumulative distribution of consensus values was computed to further calculate the proportion of ambiguously clustered pairs (PAC) to evaluate the stability of the individual cluster sizes.[10] Based on the stability measure and the consensus matrices, the dimensionally reduced selected fMRI volumes were clustered into four (using the right temporo-parietal junction [rTPJ] as seed) different states (CAPs) using the *k*-means algorithm. The individual CAPs were then reconstructed back by multiplying the PC scores with the transposed eigenvectors and adding back the mean. The CAPs were subsequently spatially *z*-scored, representing the distinct rTPJ CAPs with positive and negative contributions.[7]

For FND patients, we assigned individual frames to one of the CAPs derived from HC through a matching process. For each frame, the largest spatial correlation with each CAP was calculated. This correlation was then compared to the distribution of spatial correlations for frames from HC assigned to the same CAP. A threshold set at the 5th percentile of this distribution was used as the cutoff for assignment: if a frame's correlation exceeded this threshold, it was assigned to the corresponding CAP; otherwise, it was marked as "unassigned". This ensures that only frames with sufficient spatial similarity to a CAP were included in the analysis. In total, 14.5% of frames were classified as one of the CAPs derived from HC, while 1.54% of frames were marked as "unassigned.

rTPJ CAPs derived from healthy controls

Supplementary Figure 2. Stability measure (1 – PAC). To assess whether a certain cluster number k is good, two given data points should consistently be clustered together or in different clusters across folds. The cumulative distribution of consensus values across all pairs of data points can be computed, which gives a quantification of the goodness of fit. We refer to this distribution as $P_k(c)\ with\ c \in [0,1]$. From this, the proportion of ambiguously clustered pairs (PAC) can be computed[10] as $PAC_k = \sum_{C=c_T}^{1-c_T} P_k(c)$, with c_T a threshold consensus value above which an assignment is judged as not sufficiently homogeneous across folds, and k the cluster number. A lower PAC thus represents a more robust cluster number. The stability measure is then derived as 1 – PAC, and therefore, greater values represent more robust clusters. The individual bars (coloured) reflect the different choices for the threshold c_T. Four CAPs were preferred over two which is trivial. Four CAPs were preferred over three CAPs as it seems that two of the four CAPs were merged when clustering into three CAPs, which might explain the more fragmented expression of this CAP ($CAP1_{rTPJ}$, see below) due to its coexistence with HC-derived $CAP2_{rTPJ}$ and $CAP3_{rTPJ}$ reported in the main text.

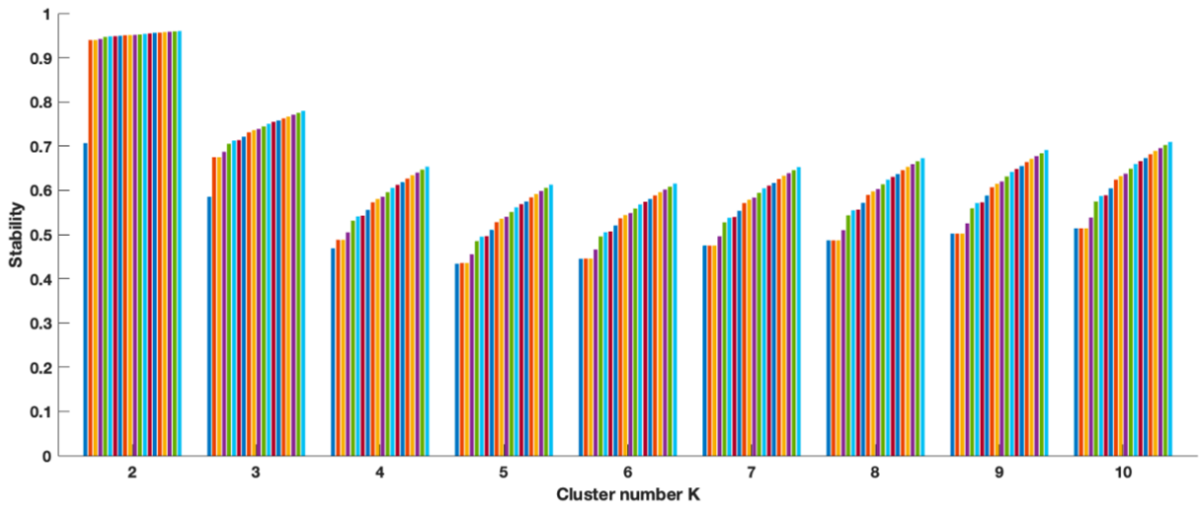

Supplementary Figure 3. Consensus matrices. The consensus matrices C_k for a given number of clusters k, which summarize consensus values across all pairs of data points, are calculated by averaging, for each entry, over all folds where two data points jointly entered the computations. A cluster is considered stable, when two arbitrary data points are continuously clustered together, as represented by crisp boundaries in the consensus matrix.

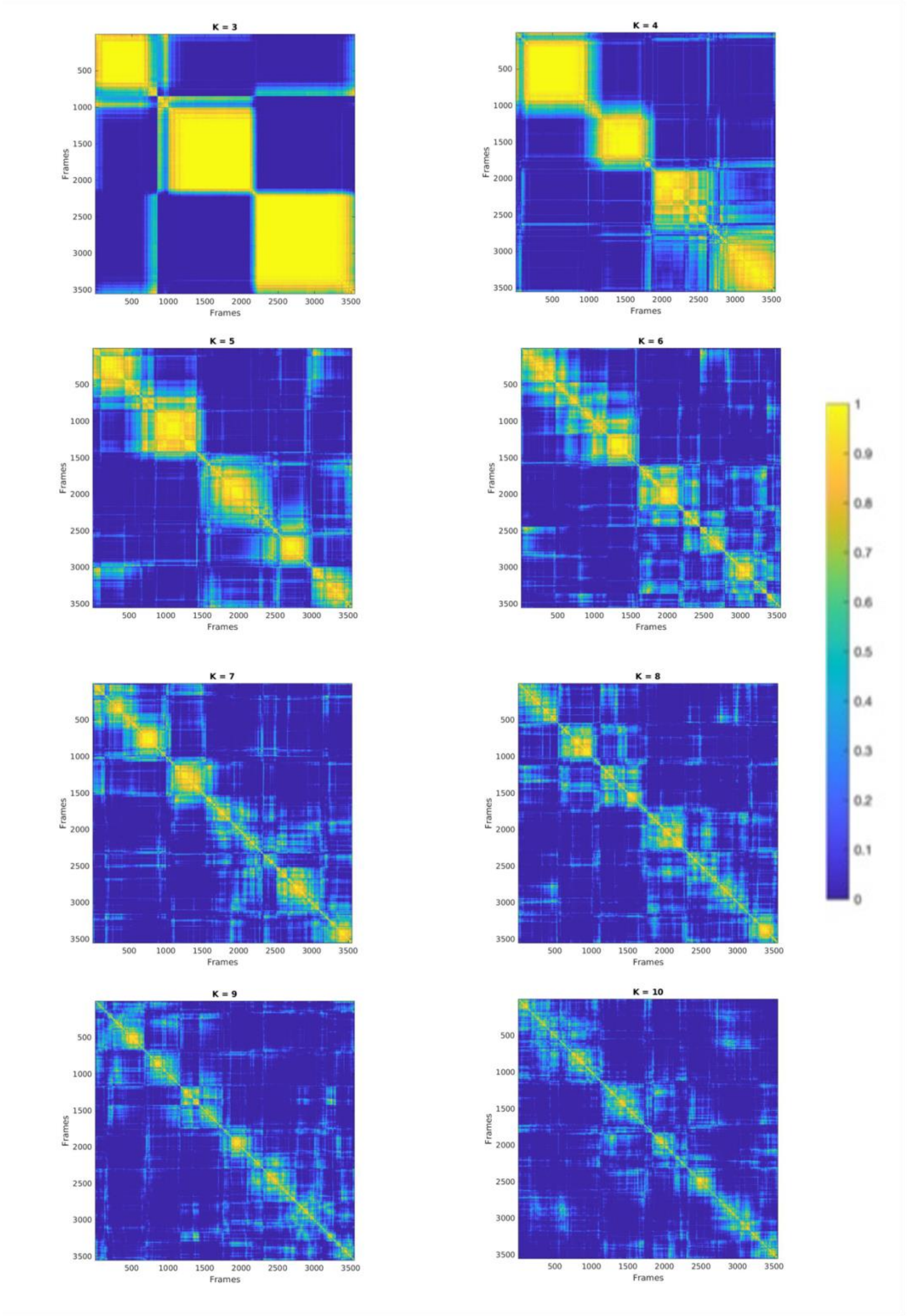

rTPJ CAPs in second most stable cluster in healthy controls

Adjusted for age, sex, number of discarded volumes, psychotropic medication, depression and anxiety scores, group comparisons of temporal characteristics revealed a significant group difference in entries for factors *state* ($F(2,336)=27.0$, $P<0.0001$), as well as in duration for the factors *group* ($F(1,336)=4.7$, $P=0.03$) and *state* $F(2,336)=5.5$, $P=0.004$). Post-hoc multiple comparisons revealed that no-FW patients entered $CAP1_{rTPJ}$ less frequently than HC ($P_{FDR}<0.0001$) and entered $CAP3_{rTPJ}$ more often than HC ($P_{FDR}=0.02$).

Supplementary Figure 4. Co-activation patterns (CAPs) obtained for the second most stable cluster number (*K* = 3), based on rTPJ seed activation of healthy controls. Three CAPs were detected. CAPs were z-scored and only the 15% most positive and 15% most negative contributions are represented in colour ($z = \pm 1.04$), with red representing positive contributions and blue negative contributions. Locations are displayed in Montreal Neurological Institute (MNI) standard space coordinates. Abbreviations: rTPJ = right temporo-parietal junction, HC = healthy controls, Cont = executive control, Default = default mode, DorsAttn = dorsal attention, Sal/VenAttn = salience/ventral attention, SomMot = somatomotor, TempPar = temporoparietal, VisCen = central visual, VisPer = peripheral visual

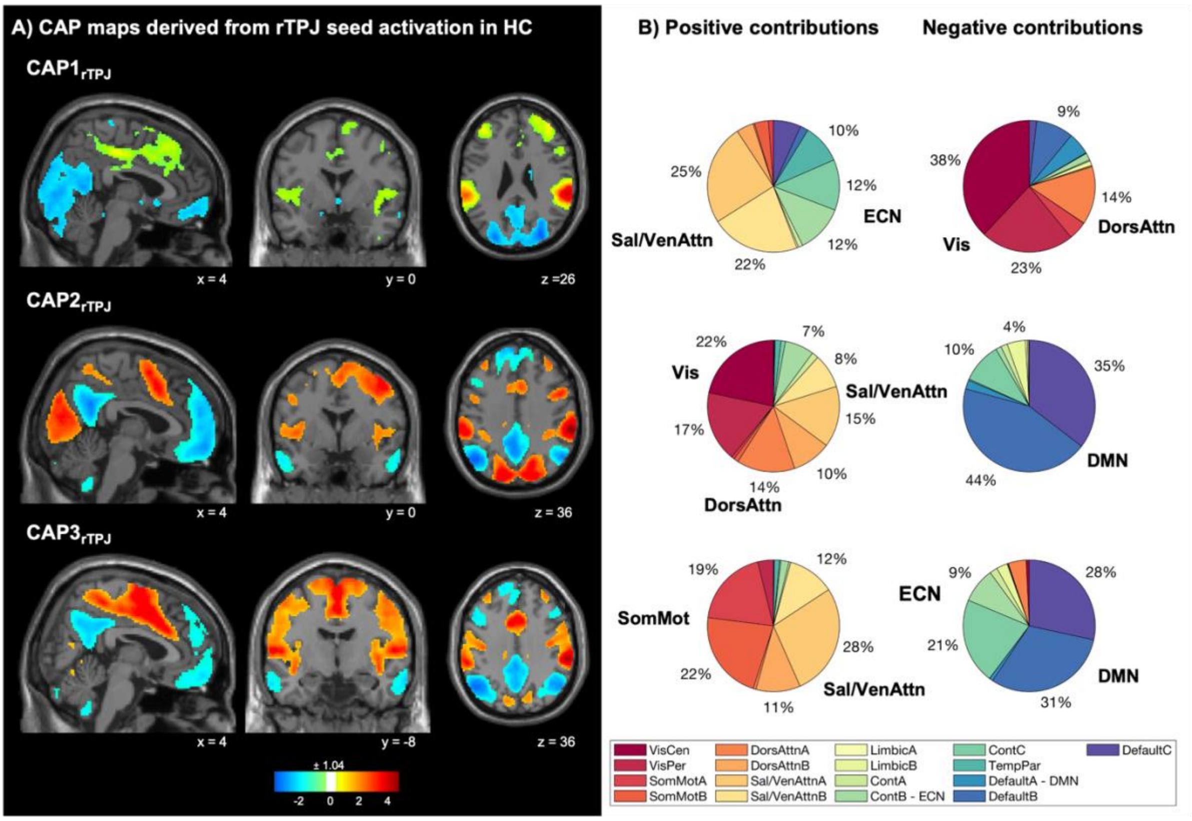

rTPJ CAPs derived from FND patients

Supplementary Figure 5. Stability measure (1 – PAC). To assess whether a certain cluster number k is good, two given data points should consistently be clustered together or in different clusters across folds. The cumulative distribution of consensus values across all pairs of data points can be computed, which gives a quantification of the goodness of fit. We refer to this distribution as $P_k(c)\ with\ c \in [0,1]$. From this, the proportion of ambiguously clustered pairs (PAC) can be computed[10] as $PAC_k = \sum_{C=c_T}^{1-c_T} P_k(c)$, with c_T a threshold consensus value above which an assignment is judged as not sufficiently homogeneous across folds, and k the cluster number. A lower PAC thus represents a more robust cluster number. The stability measure is then derived as 1 – PAC, and therefore, greater values represent more robust clusters. The individual bars (coloured) reflect the different choices for the threshold c_T. Four CAPs were preferred over two which is trivial. Four CAPs were preferred over three CAPs as it seems that two of the four CAPs were merged when clustering into three CAPs, which might explain the more fragmented expression of this CAP ($CAP1_{rTPJ}$ see below) due to its coexistence with FND-derived $CAP2_{rTPJ}$ and $CAP4_{rTPJ}$ reported in the main text. Four CAPs were preferred over five, as consensus matrices showed more consistent clustering for $K = 4$ (as indicated by more crisp boundaries).

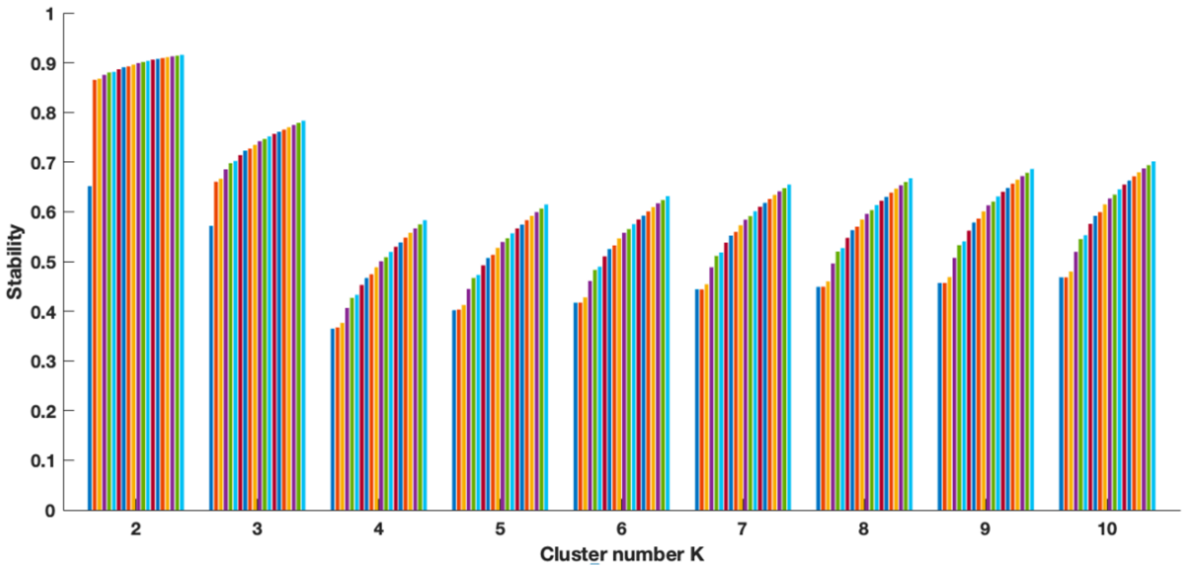

Supplementary Figure 6. Consensus matrices. The consensus matrices C_k for a given number of clusters k, which summarize consensus values across all pairs of data points, are calculated by averaging, for each entry, over all folds where two data points jointly entered the computations. A cluster is considered stable, when two arbitrary data points are continuously clustered together, as represented by crisp boundaries in the consensus matrix.

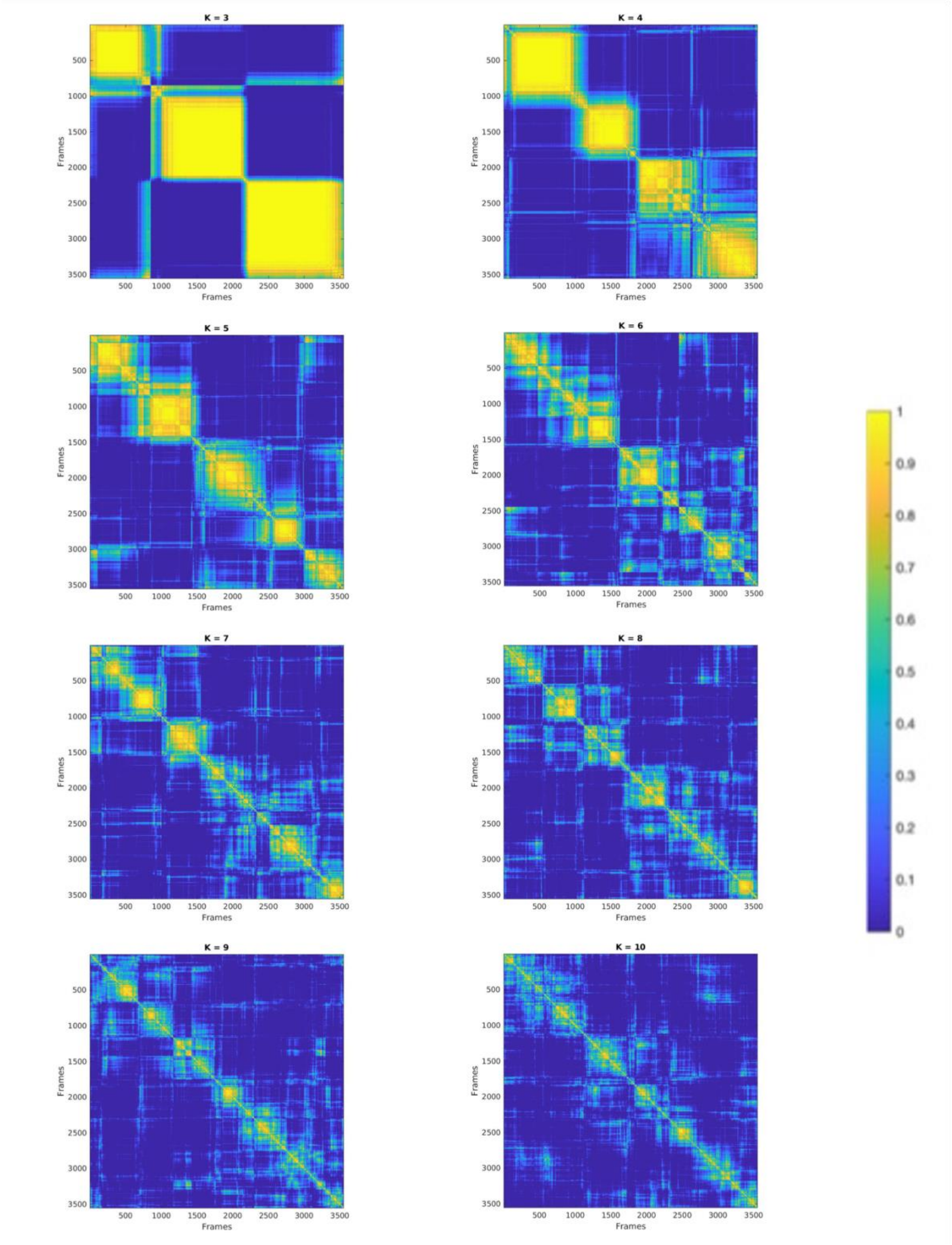

rTPJ CAPs in second most stable cluster in FND patients

Adjusted for age, sex, number of discarded volumes, psychotropic medication, depression and anxiety scores, group comparisons of temporal characteristics revealed a significant group (FW vs no-FW) difference in entries for factors *state* ($F(2,162)=25.0$, $P=0.0008$), as well as in duration for the factors *group* ($F(1,162)=4.2$, $P=0.04$). Post-hoc multiple comparisons revealed that FW patients had a longer duration of $CAP3_{rTPJ}$ compared to no-FW patients ($P_{FDR}=0.03$) and entered $CAP3_{rTPJ}$ more often than HC ($P_{FDR}=0.02$).

Supplementary Figure 7. Co-activation patterns (CAPs) obtained for the second most stable cluster number (*K* = 3), based on rTPJ seed activation of FND patients. Three CAPs were detected. CAPs were z-scored and only the 15% most positive and 15% most negative contributions are represented in colour ($z = \pm 1.04$), with red representing positive contributions and blue negative contributions. Locations are displayed in Montreal Neurological Institute (MNI) standard space coordinates. Abbreviations: rTPJ = right temporo-parietal junction, FND = functional neurological disorders, Cont = executive control, Default = default mode, DorsAttn = dorsal attention, Sal/VenAttn = salience/ventral attention, SomMot = somatomotor, TempPar = temporoparietal, VisCen = central visual, VisPer = peripheral visual

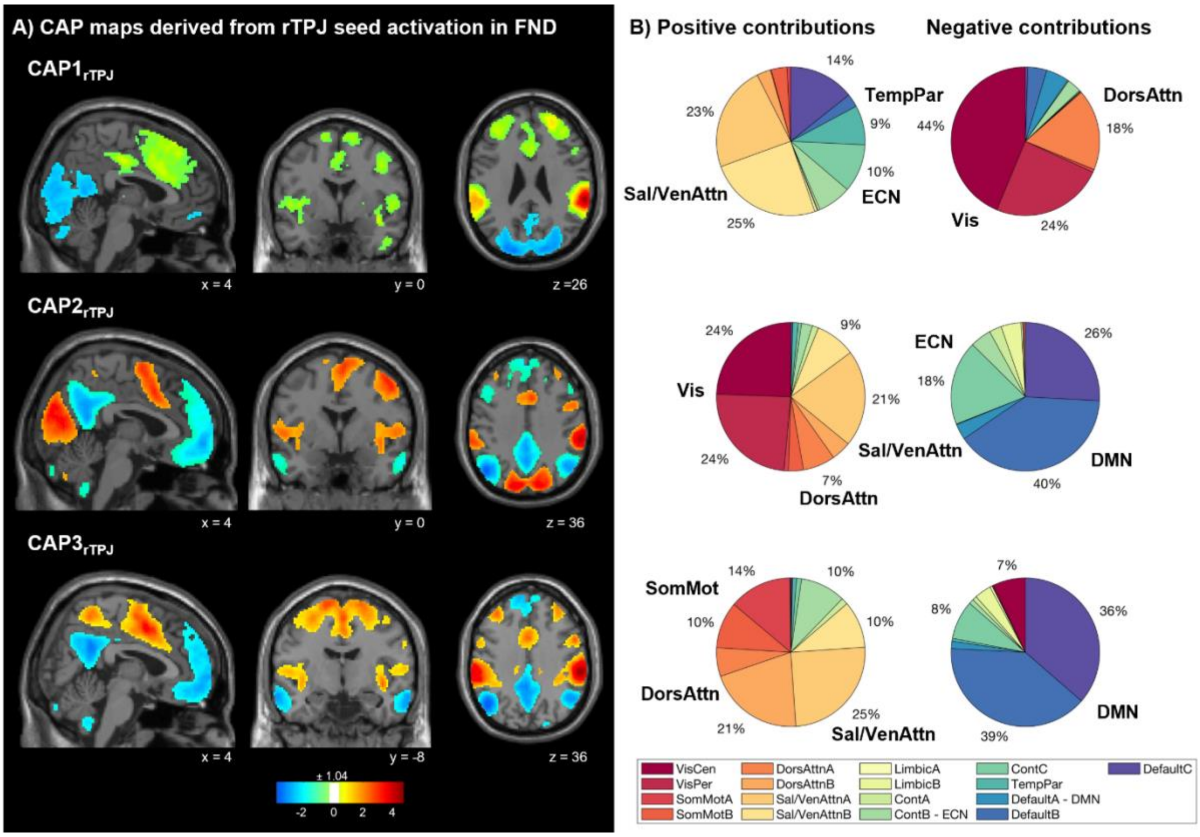

lTPJ CAPs as control seed in healthy controls

To address the question on the specificity of the findings on the rTPJ, we analysed additionally CAPs derived from the left TPJ (lTPJ) as seed. Correspondingly, the left TPJ seed was defined as a sphere of 10mm centred at Montreal Neurological Institute (MNI) coordinates [-62 -34 30]. All the other computations were repeated equally as described in the main manuscript and the supplementary material. The CAPs derived from HC using rTPJ as seed showed high voxel overlap with those derived from HC using lTPJ as seed: $CAP1_{rTPJ}$ (97%), $CAP2_{rTPJ}$ (92%), $CAP3_{rTPJ}$ (88%), and $CAP4_{rTPJ}$ (95%).

Adjusted for age, sex, number of discarded volumes, psychotropic medication, depression and anxiety scores, group comparisons of temporal characteristics revealed a significant group difference in entries for factors *state* ($F(2,445)=22.3$, $P<0.0001$), as well as in duration for the factors *group* ($F(1,445)=3.1$, $P=0.04$) and *state* $F(2,445)=6.5$, $P=0.0002$). Post-hoc multiple comparisons revealed that FW patients entered $CAP1_{lTPJ}$ more frequently than HC ($P_{FDR}=0.03$) and entered $CAP3_{lTPJ}$ less often than HC ($P_{FDR}=0.03$); while no-FW patients entered $CAP2_{lTPJ}$ less frequently than HC ($P_{FDR}=0.03$) and entered $CAP3_{lTPJ}$ more often than HC ($P_{FDR}=0.01$). There were no significant different in terms of duration.

Supplementary Figure 8. Co-activation pattern (CAP) maps based on lTPJ seed activation derived from healthy controls (HC). (A) Four CAPs were detected. CAPs were z-scored and only the 15% most positive and 15% most negative contributions are represented in colour (z = ± 1.04), with red representing positive contributions and blue negative contributions. Locations are displayed in Montreal Neurological Institute (MNI) standard space coordinates. **(B)** Pie charts illustrating the percentage of positive and negative contributions within the 17 resting-state networks according to the convention of Yeo[11]. Seed voxels were removed. Abbreviations: rTPJ = right temporo-parietal junction, HC = healthy controls, Cont = executive control, Default = default mode, DorsAttn = dorsal attention, Sal/VenAttn = salience/ventral attention, SomMot = somatomotor, TempPar = temporoparietal, VisCen = central visual, VisPer = peripheral visual.

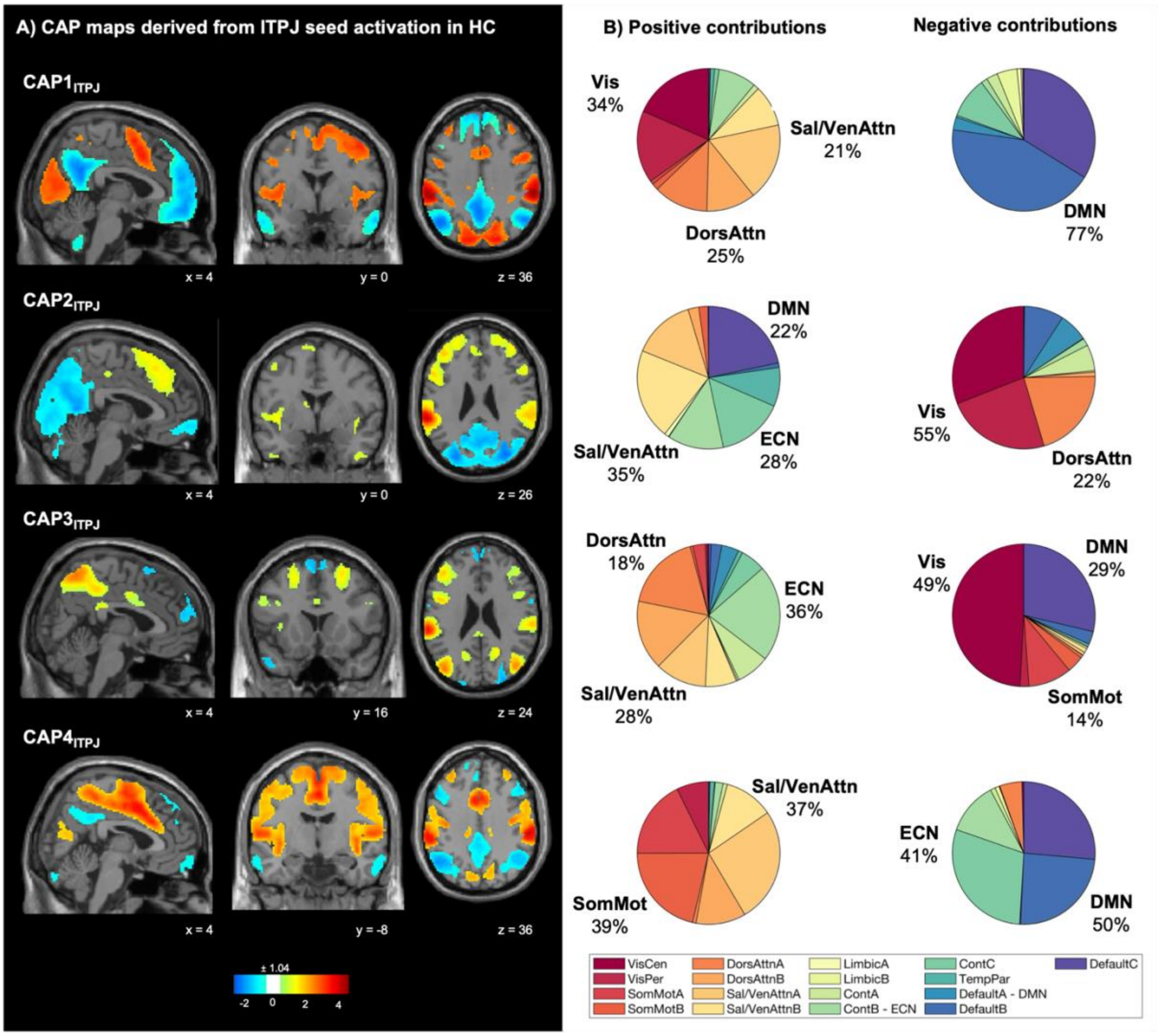

Supplementary Figure 9. Stability measure (1 – PAC). To evaluate cluster quality, data points should consistently group together or separately across folds. The proportion of ambiguously clustered pairs (PAC) quantifies this, with lower PAC values indicating more robust clusters. The stability measure, calculated as *1 – PAC*, reflects cluster robustness, with higher values being better. Four CAPs were preferred as they provided more consistent clustering than three (where merging occurred) and five (which showed fewer crisp boundaries in consensus matrices).

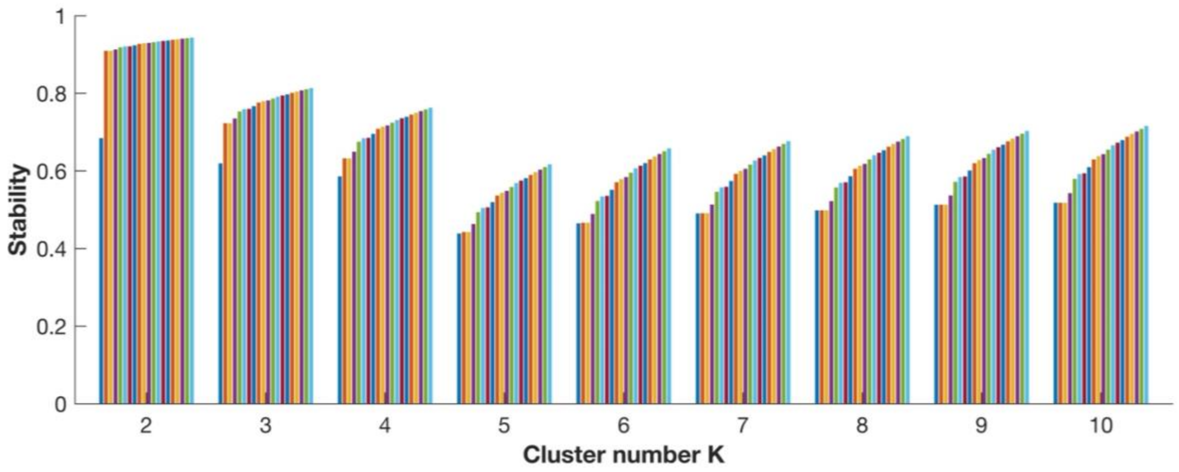

Supplementary Figure 10. Consensus matrices. The consensus matrices C_k for a given number of clusters k, which summarize consensus values across all pairs of data points, are calculated by averaging, for each entry, over all folds where two data points jointly entered the computations. A cluster is considered stable, when two arbitrary data points are continuously clustered together, as represented by crisp boundaries in the consensus matrix.

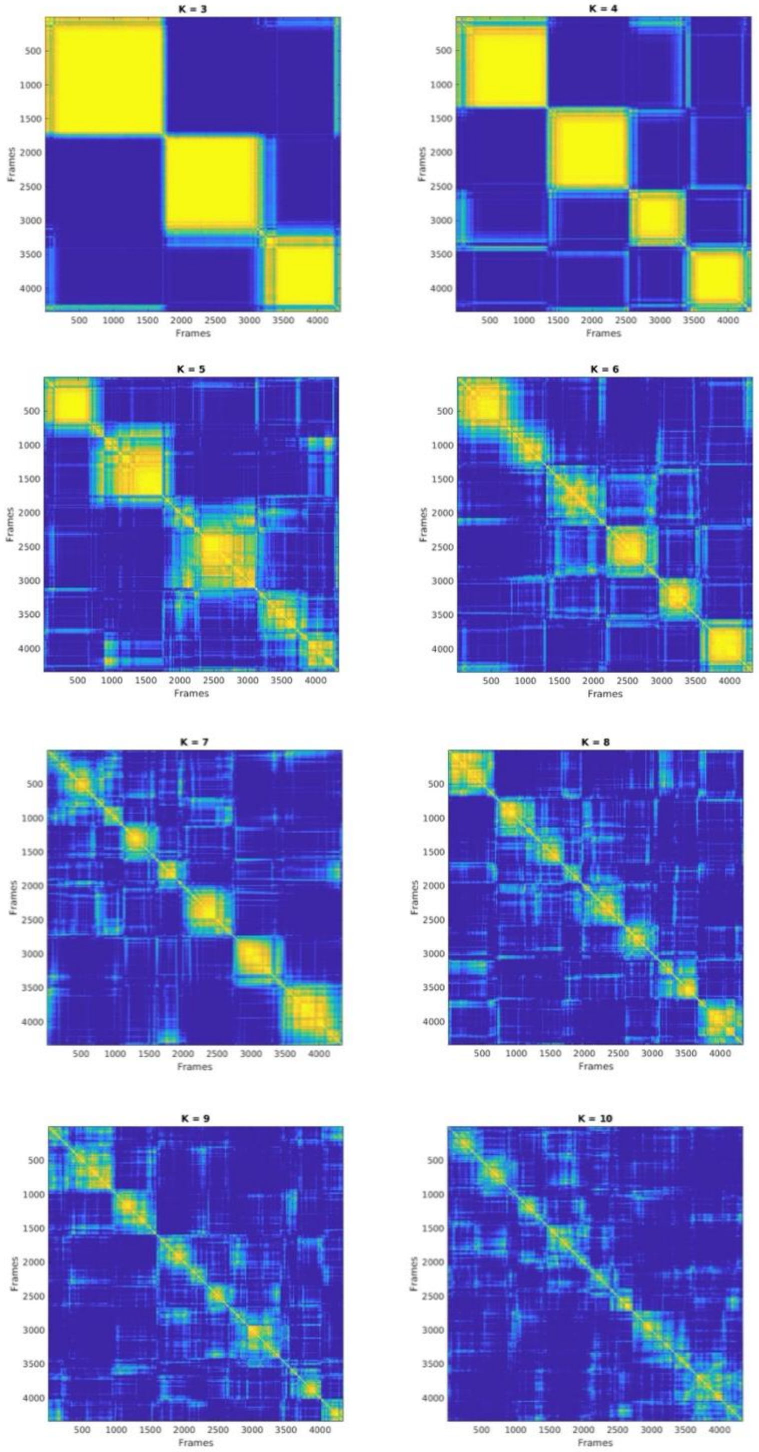

lTPJ CAPs as control seed in FND patients

To address the question on the specificity of the findings on the rTPJ, we analysed additionally CAPs derived from the left TPJ (lTPJ) as seed. Correspondingly, the left TPJ seed was defined as a sphere of 10mm centred at Montreal Neurological Institute (MNI) coordinates [-62 -34 30]. All the other computations were repeated equally as described in the main manuscript and the supplementary material. The CAPs derived from FND using rTPJ as seed showed a relatively high voxel overlap with those derived from FND using lTPJ as seed: $CAP1_{rTPJ}$ (95%), $CAP2_{rTPJ}$ (77%), $CAP3_{rTPJ}$ (91%), and $CAP4_{rTPJ}$ (90%).

Adjusted for age, sex, number of discarded volumes, psychotropic medication, depression and anxiety scores, group comparisons of temporal characteristics revealed a significant group (FW vs no-FW) difference in entries for factors *state* ($F(3,218)=19.0$, $P<0.0001$). Post-hoc multiple comparisons revealed no significant differences.

Supplementary Figure 11. Co-activation pattern (CAP) maps based on lTPJ seed activation derived from FND patients. (A) Four CAPs were detected. CAPs were z-scored and only the 15% most positive and 15% most negative contributions are represented in colour (z = ± 1.04), with red representing positive contributions and blue negative contributions. Locations are displayed in Montreal Neurological Institute (MNI) standard space coordinates. **(B)** Pie charts illustrating the percentage of positive and negative contributions within the 17 resting-state networks according to the convention of Yeo[11]. Seed voxels were removed. Abbreviations: rTPJ = right temporo-parietal junction, FND = functional neurological disorders, Cont = executive control, Default = default mode, DorsAttn = dorsal attention, Sal/VenAttn = salience/ventral attention, SomMot = somatomotor, TempPar = temporoparietal, VisCen = central visual, VisPer = peripheral visual.

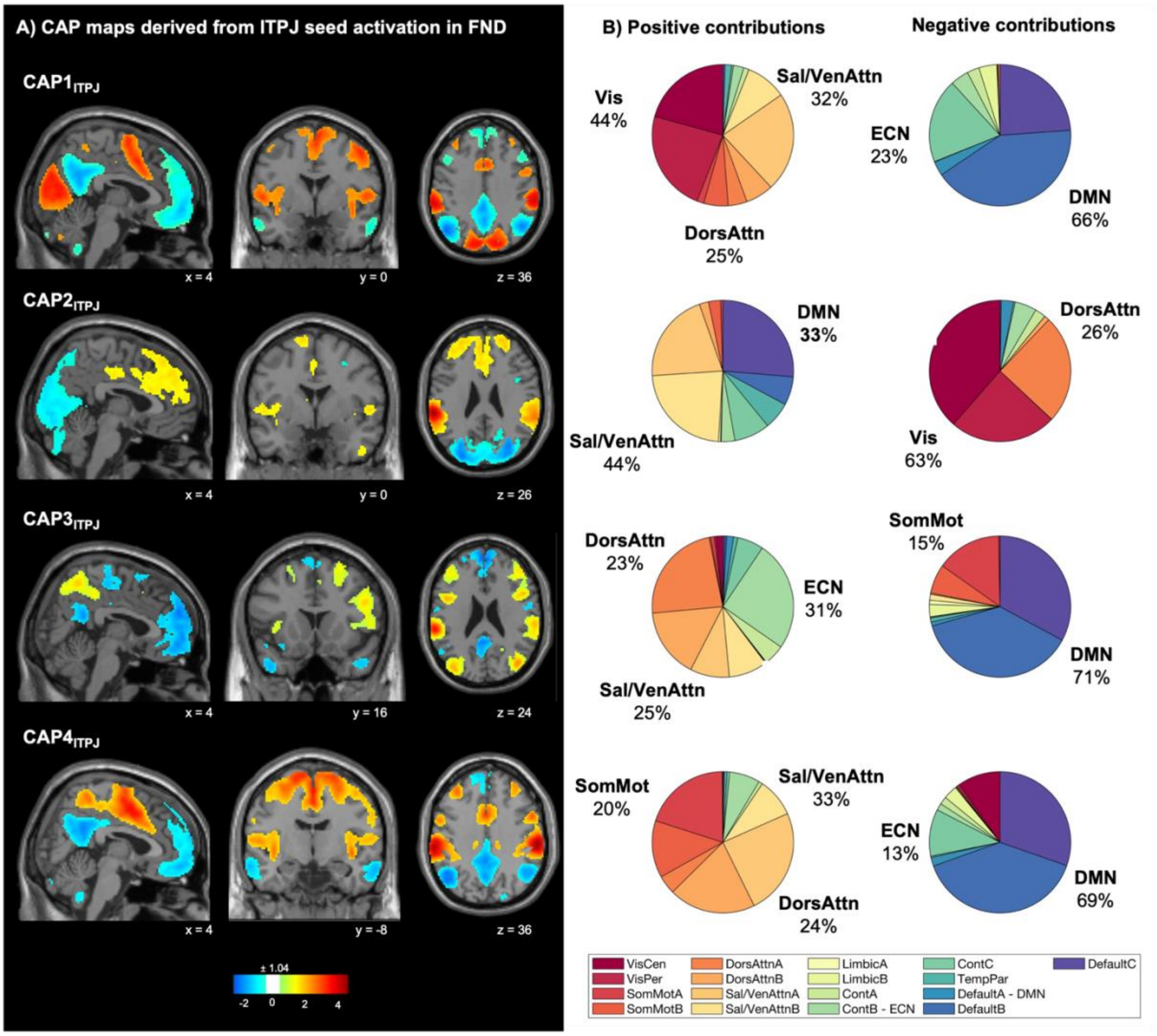

Supplementary Figure 12. Stability measure (1 – PAC). To evaluate cluster quality, data points should consistently group together or separately across folds. The proportion of ambiguously clustered pairs (PAC) quantifies this, with lower PAC values indicating more robust clusters. The stability measure, calculated as *1 – PAC*, reflects cluster robustness, with higher values being better.

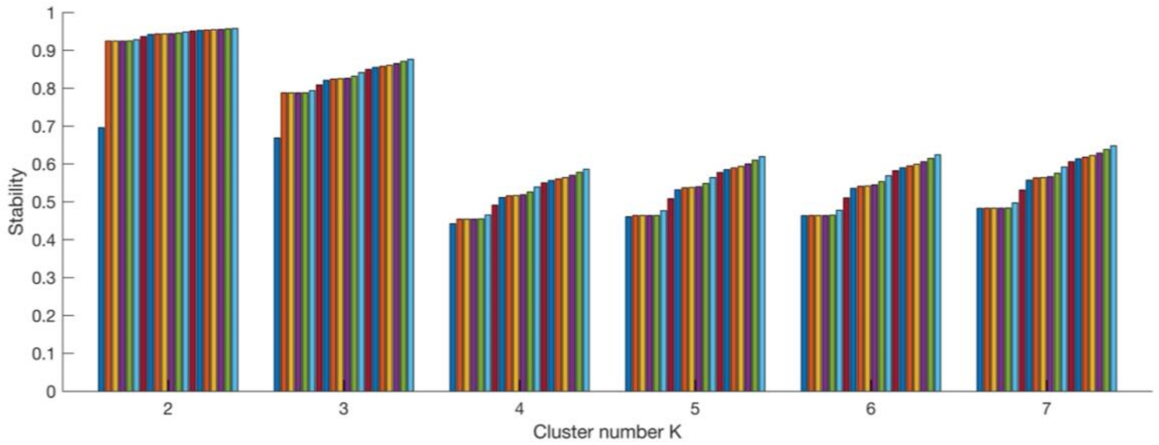

Supplementary Figure 13. Consensus matrices. The consensus matrices C_k for a given number of clusters k, which summarize consensus values across all pairs of data points, are calculated by averaging, for each entry, over all folds where two data points jointly entered the computations. A cluster is considered stable, when two arbitrary data points are continuously clustered together, as represented by crisp boundaries in the consensus matrix.

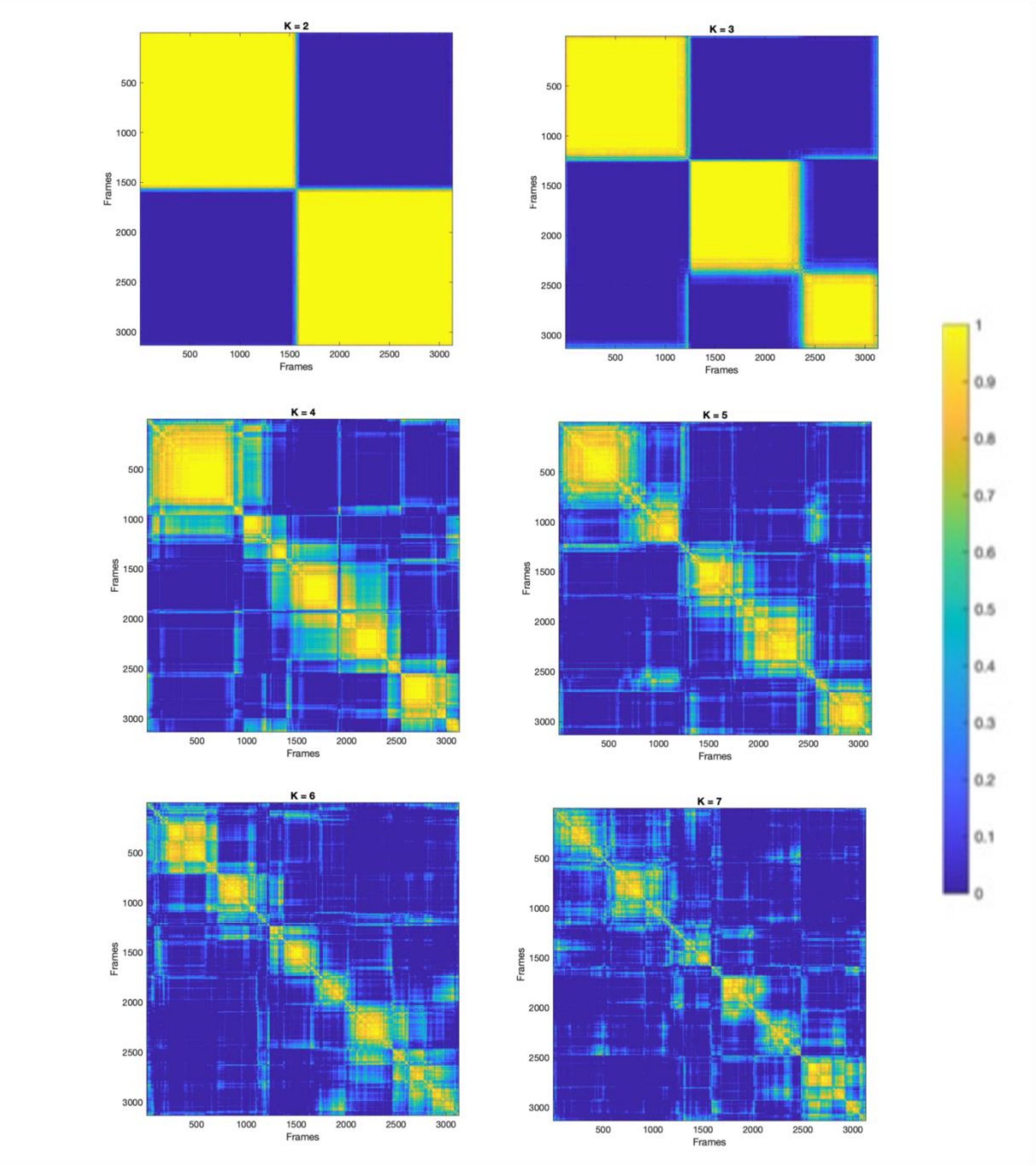

References

1. Gallichan D, Marques JP, Gruetter R. Retrospective correction of involuntary microscopic head movement using highly accelerated fat image navigators (3D FatNavs) at 7T. *Magn Reson Med*. 2016;75(3):1030-1039. doi:10.1002/mrm.25670
2. Richiardi J, Van De Ville D, Riesen K, Bunke H. Vector space embedding of undirected graphs with fixed-cardinality vertex sequences for classification. *Proceedings - International Conference on Pattern Recognition*. Published online 2010:902-905. doi:10.1109/ICPR.2010.227
3. Weber S, Heim S, Richiardi J, Ville D Van De, Aybek S. Multi-centre classification of functional neurological disorders based on resting-state functional connectivity. 2022;35(May). doi:10.1016/j.nicl.2022.103090
4. Richiardi J, Eryilmaz H, Schwartz S, Vuilleumier P, Van De Ville D. Decoding brain states from fMRI connectivity graphs. *Neuroimage*. 2011;56(2):616-626. doi:10.1016/j.neuroimage.2010.05.081
5. Richiardi J, Gschwind M, Simioni S, et al. Classifying minimally disabled multiple sclerosis patients from resting state functional connectivity. *Neuroimage*. 2012;62(3):2021-2033. doi:10.1016/j.neuroimage.2012.05.078
6. Bolton TAW, Tuleasca C, Wotruba D, et al. TbCAPs: A toolbox for co-activation pattern analysis. *Neuroimage*. 2020;211(October 2019):116621. doi:10.1016/j.neuroimage.2020.116621
7. Liu X, Duyn JH. Time-varying functional network information extracted from brief instances of spontaneous brain activity. *Proceedings of the National Academy of Sciences*. 2013;110(11):4392-4397. doi:10.1073/pnas.1216856110
8. Weber S, Bühler J, Loukas S, et al. Transient resting-state salience-limbic co-activation patterns in functional neurological disorders. *Neuroimage Clin*. 2024;41:103583. doi:10.1016/j.nicl.2024.103583
9. Weber S, Jungilligens J, Aybek S, Popkirov S. Locus coeruleus co-activation patterns at rest show higher state persistence in patients with dissociative seizures: A Pilot Study. *Epilepsia Open*. Published online October 7, 2024. doi:10.1002/epi4.13050
10. Şenbabaoğlu Y, Michailidis G, Li JZ. Critical limitations of consensus clustering in class discovery. *Sci Rep*. 2014;4(1):6207. doi:10.1038/srep06207
11. Yeo Th, Krienen FM, Sepulcre J, et al. The organization of the human cerebral cortex estimated by intrinsic functional connectivity. *J Neurophysiol*. 2011;106(3):1125-1165. doi:10.1152/jn.00338.2011
